# G-quadruplex stabilization via small molecules as a potential anti-cancer strategy

**DOI:** 10.1186/s11658-026-00890-3

**Published:** 2026-03-13

**Authors:** Chunshuang Li, Jialian Liu, Ruoxi Wang

**Affiliations:** 1 Center for Cell Structure and Function, Key Laboratory of Animal Resistance Biology of Shandong Province, Collaborative Innovation Center of Cell Biology in Universities of Shandong, College of Life Sciences, Shandong Normal University, Jinan, 250358 China; 2 School of Chemistry and Life Science, Changchun University of Technology, Changchun, 130012 China

**Keywords:** G-quadruplex, Telomere maintenance, Gene regulation, Genome instability, Small-molecule ligands, Cancer therapeutics

## Abstract

G-quadruplexes (G4s) are noncanonical nucleic acid secondary structures formed by guanine-rich DNA or RNA sequences, predominantly located at telomeric ends and within the promoter regions of oncogenes. These structures play essential roles in maintaining telomere stability, regulating DNA replication, and modulating gene transcription and translation. Stabilization of G4 structures can induce cellular senescence and apoptosis while suppressing oncogene expression, positioning them as highly promising therapeutic targets in anticancer research. In recent years, a variety of small-molecule G4 ligands have been developed, many of which exhibit potent antitumor activity by selectively stabilizing G4s. Targeting G4s has thus emerged as a cutting-edge strategy in cancer therapy, offering new avenues for precision medicine. This review provides a comprehensive overview of G4 structure and function, highlights recent progress in the development of G4-targeting ligands, and discusses their therapeutic potential in oncology. Our goal is to offer insights into the design and application of G4-targeted agents for future anticancer drug development.

**Graphical abstract:**

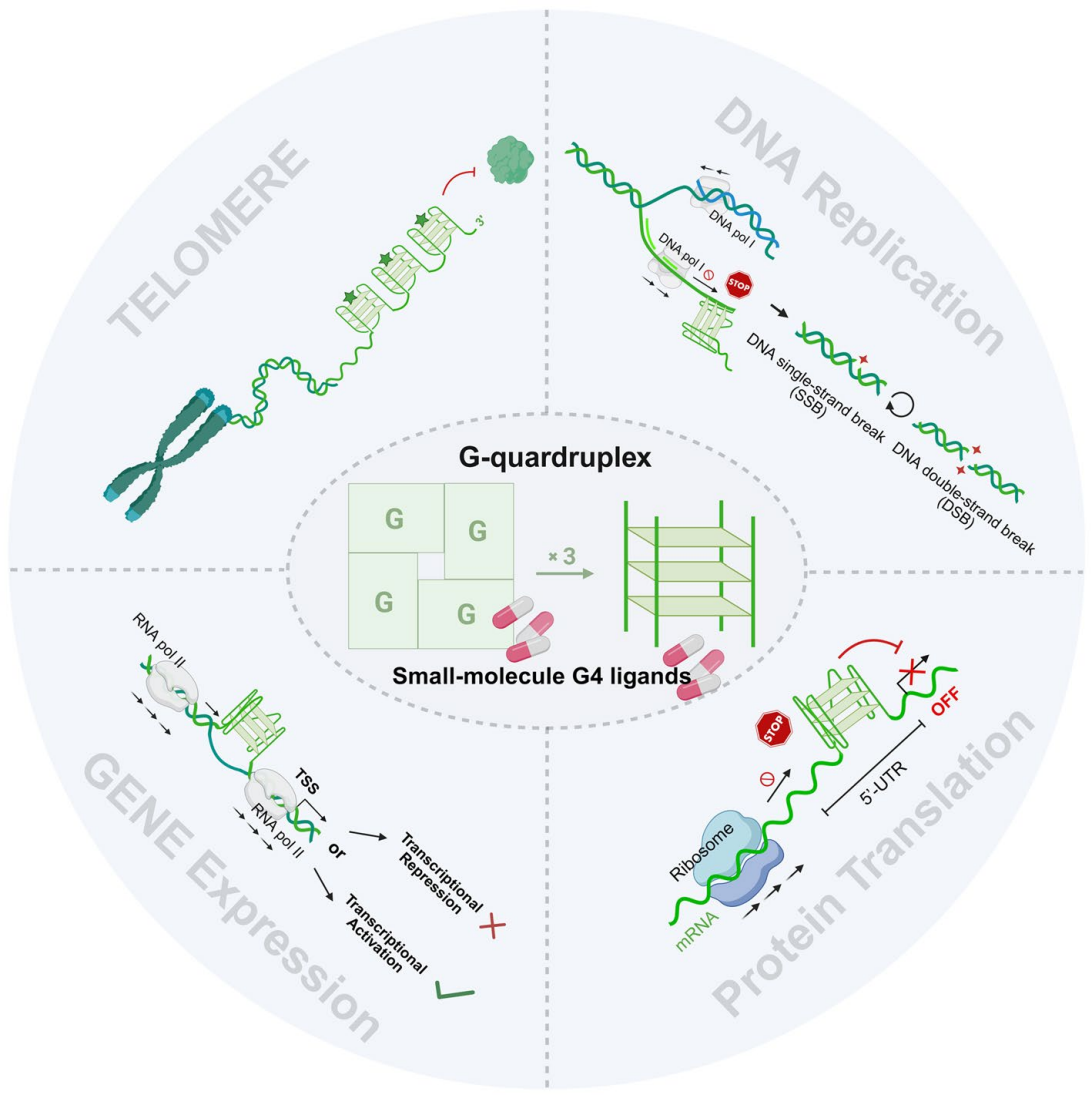

## Introduction

DNA, as the genetic material, predominantly exists in vivo as the canonical Watson–Crick double helix, characterized by T:A and C:G base pairing. Similarly, RNA secondary structures are largely stabilized by Watson–Crick interactions. Beyond these classical base pairs, numerous alternative pairing modes exist, providing the foundation for diverse and stable structural motifs [1]. One such motif is the G-quadruplex (G4), a noncanonical nucleic acid secondary structure formed by guanine-rich DNA or RNA sequences [2]. The presence of G4 structures within cells—particularly in cancer cells—has been confirmed using G4-specific BG4 antibodies and fluorescent probes [3, 4]. Accumulating evidence indicates that G4 structures are involved in essential genomic processes, including DNA replication, gene transcription, translation, and epigenetic regulation [2, 5]. Moreover, G4s play regulatory roles in malignant transformation and cancer progression, highlighting their potential as therapeutic targets [6]. In recent years, numerous small-molecule G4 ligands have been developed to induce and stabilize G4 formation, disrupt oncogenic signaling, suppress oncogene transcription and expression, and inhibit telomerase activity, thereby impeding tumor cell proliferation and promoting apoptosis [7, 8]. In this review, we summarize the structural features and biological functions of G4s, and discuss current therapeutic strategies that target G4 structures for cancer treatment.

## Structure and function of the G-quadruplex

### G-quadruplex structure

**Fig. 1:**
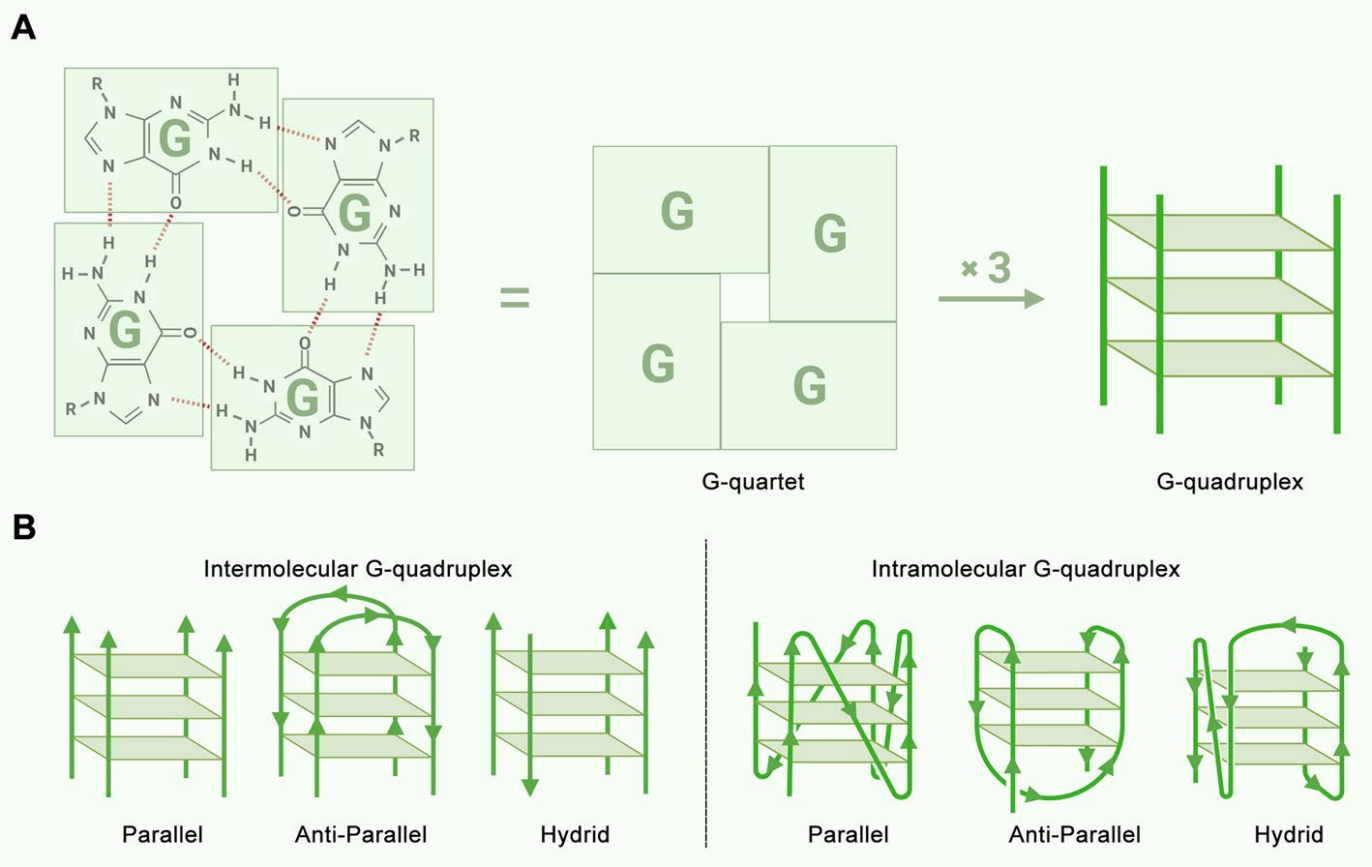
G-quadruplex (G4) structures. **A** Schematic representation of a G-quartet formed via Hoogsteen hydrogen bonding among four guanine bases, and the assembly of a G4 structure through the stacking of three G-quartets. **B** Representative topological conformations of G4 structures, including intermolecular and intramolecular G-quadruplexes, with parallel, antiparallel, and hybrid folding patterns

In 1910, Bang first reported that concentrated guanylic acid solutions could form gels. Later, in 1962, Gellert et al. revealed by X-ray crystallography that the gelatinous material formed by guanosine monophosphate (GMP) consisted of guanine tetramers [9]. These tetramers, termed G-quartets (or G-tetrads), adopt a square planar configuration stabilized by Hoogsteen hydrogen bonds among four guanine bases [10] (Fig. 1A). Studies with synthetic oligoguanine sequences initially demonstrated that G4 motifs can exhibit high thermodynamic stability, resisting denaturation even under low-salt conditions and showing limited hybridization with oligocytosine strands [11]. However, accumulating evidence from more recent studies indicates that C-rich complementary strands are capable of invading and destabilizing preformed G4 structures, underscoring the dynamic and context-dependent nature of G4 folding in vitro [12–14]. X-ray crystallography has confirmed that G-quartets can stack to form higher-order architectures, giving rise to the unique G4 structure [15]. G4s are stabilized by π–π stacking interactions between G-quartet planes, with thermodynamic stability increasing as the number of stacked quartets grows (Fig. 1A). Monovalent cations, particularly K^+^, followed by Na^+^, NH_4_^+^, and Li^+^, play a critical role in G4 stabilization, with K^+^ being the most effective due to its optimal ionic radius, which both reduces the solvation energy compared with Na^+^ and fits snugly within the central cavity of the G-quartets, coordinating with carbonyl oxygens [12, 16–18].

G4s exhibit remarkable structural polymorphism. They can form via intramolecular folding of a single nucleic acid strand (intramolecular G4) or through association of two or more strands (intermolecular G4) [19] (Fig. 1B). Intramolecular G4s, independent of strand concentration, are the predominant form in vivo, whereas intermolecular G4s require higher strand concentrations and display increasing structural complexity [20]. Each G-quartet layer maintains 5′ → 3′ strand polarity, giving rise to three major topologies based on strand orientation: (1) parallel, with all four strands running in the same direction; (2) antiparallel, with two strands in opposite directions; and (3) hybrid, where three strands align in one direction and the fourth in the opposite direction (Fig. 1B). Parallel G4s typically feature three propeller-type loops connecting adjacent quartets, with all guanines adopting *anti*-conformation. Antiparallel G4s form either chair-type topologies with lateral loops or basket-type arrangements with lateral and diagonal loops. Hybrid G4s often combine propeller, lateral, and diagonal loops [12, 19, 21, 22]. Loop length and composition further modulate G4 stability; shorter loops generally confer higher stability and are more common in biologically relevant G4s, although some functional G4s tolerate unusually long loops [23].

In vitro, G4s preferentially form at sequences matching the consensus motif G ≥ 3N_1–7_G ≥ 3N_1–7_G ≥ 3N_1–7_G ≥ 3 [19, 24]. Computational predictions and deep-sequencing approaches have identified over 700,000 potential G4-forming sequences (pG4s or PQSs) in the human genome [24–26]. These sequences are non-randomly distributed, with strong enrichment at telomeres and gene promoters. Genome-wide analyses revealed nearly 20,000 PQSs in promoters, mostly concentrated within 1 kb upstream and 500 nt downstream of transcription start sites (TSSs) [27]. Additional G4 motifs have been detected in DNA replication origins [28], noncoding RNAs [29], 5′-untranslated regions (5′-UTRs) [30], and ribosomal DNA [31, 32]. G4s are also present in human mitochondrial DNA (mtDNA), contributing to mitochondrial genome instability [33, 34].

### Detection and visualization of G-quadruplex structures

Given the structural diversity and widespread genomic distribution of G-quadruplexes described above, reliable methods for their detection and visualization are essential for elucidating their biological relevance. In recent years, a variety of complementary approaches have been developed to identify G4 structures in vitro, in cells, and at the genome-wide level.

In vitro biophysical analyses are widely employed to validate G4 formation, topology, and stability using synthetic oligonucleotides. Spectroscopic techniques such as circular dichroism (CD) spectroscopy provide insights into G4 topology, whereas nuclear magnetic resonance (NMR) spectroscopy and X-ray crystallography offer atomic-level structural information [35, 36]. Thermal stability is commonly assessed by ultraviolet (UV) melting assays, which measure the melting temperature (*T*_m_) of G4s in the presence of stabilizing monovalent cations such as K^+^ or Na^+^. In addition, native polyacrylamide gel electrophoresis (PAGE) and size-exclusion chromatography (SEC) are frequently used to evaluate G4 conformation and molecular stoichiometry [37].

Cellular imaging approaches enable visualization of G4 structures in fixed or living cells. Immunofluorescence using the G4-specific BG4 antibody has been extensively applied to detect DNA G4s in fixed cells[3]. Alternatively, small-molecule fluorescent probes, such as *N*-methyl mesoporphyrin IX (NMM), selectively bind G4s and emit fluorescence upon interaction. When combined with fluorescence lifetime imaging microscopy (FLIM), these probes allow monitoring of G4 dynamics in live cells. Chemical probing strategies that target single-stranded regions indicative of G4 folding further complement imaging-based approaches [38–40].

Genome-wide mapping strategies provide high-throughput identification of G4-forming loci across genomes and transcriptomes. Sequencing-based methods, including G4-seq for DNA and rG4-seq for RNA, exploit polymerase stalling at G4 structures to map their genomic distribution. More recently, cleavage under targets and tagmentation (CUT&Tag) coupled with BG4 antibodies has enabled in situ profiling of protein-associated G4s [20, 25]. Chemical pull-down approaches, such as G4RP-seq, use biotinylated G4 ligands to enrich and identify RNA G4s. In parallel, bioinformatic tools (e.g., G4Hunter) are widely used to predict potential G4-forming sequences, although experimental validation remains essential [41, 42]. For example, by exploiting the G4-binding domain of the helicase DHX36, Zheng Tan and colleagues engineered an artificial G4 probe that, when combined with ChIP-seq, enabled the identification of more than 100,000 G4 structures in human cells [43]. Moreover, Di Antonio et al. developed small-molecule fluorescent probes that allow real-time single-molecule tracking of G4 structures in live cells while preserving their native folding dynamics [4].

### G-quadruplex function

#### G-quadruplex in telomeres

Telomeres are nucleoprotein complexes at the ends of linear eukaryotic chromosomes, preventing degradation and end-to-end fusion, and preserving coding region integrity [44, 45]. In normal somatic cells, telomerase is usually inactive, leading to progressive telomere shortening and, when critically short, triggering DNA damage responses that induce senescence or apoptosis [46, 47]. By contrast, approximately 80–85% of cancer cells exhibit aberrant telomerase reactivation, enabling unlimited replicative potential [48]. Consequently, telomeres and their maintenance mechanisms are key therapeutic targets in cancer [49].

**Fig. 2:**
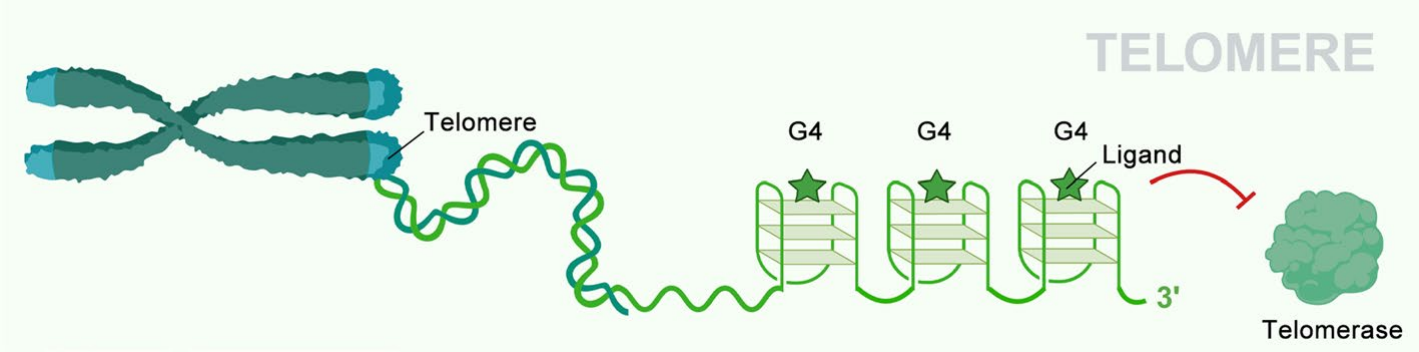
G-quadruplexes at telomeres. The G-rich overhang of telomeres can form G4 structures and stabilizing of G4s by ligands inhibit telomerase-mediated telomere elongation, promoting telomere shortening

Human telomeric DNA consists of tandem repeats of the hexameric sequence d(TTAGGG), terminating in a single-stranded 3′ overhang that is particularly enriched in pG4s [50, 51]. Telomeric DNA was shown to adopt G4 structures as early as the 1980s [52, 53]. These G4s play dual roles in telomere biology: safeguarding telomeric integrity and regulating telomere length. Under physiological conditions, the G-rich strand readily folds into G4s, particularly near the 3′ overhang where the propensity for quadruplex formation is highest [54] (Fig. 2). By forming stable higher-order structures, telomeric G4s contribute to the structural stability of chromosome ends and are involved in telomere length regulation [55].

#### G-quadruplex in gene expression

Bioinformatic analyses have revealed that G4s are widespread and highly enriched in gene promoter regions, particularly near transcription start sites [56]. Over 40% of human promoters contain at least one G4 motif [57, 58], suggesting a pivotal role in transcriptional regulation. These structures exert dual effects on transcription initiation, functioning as either enhancers or repressors.

**Fig. 3:**
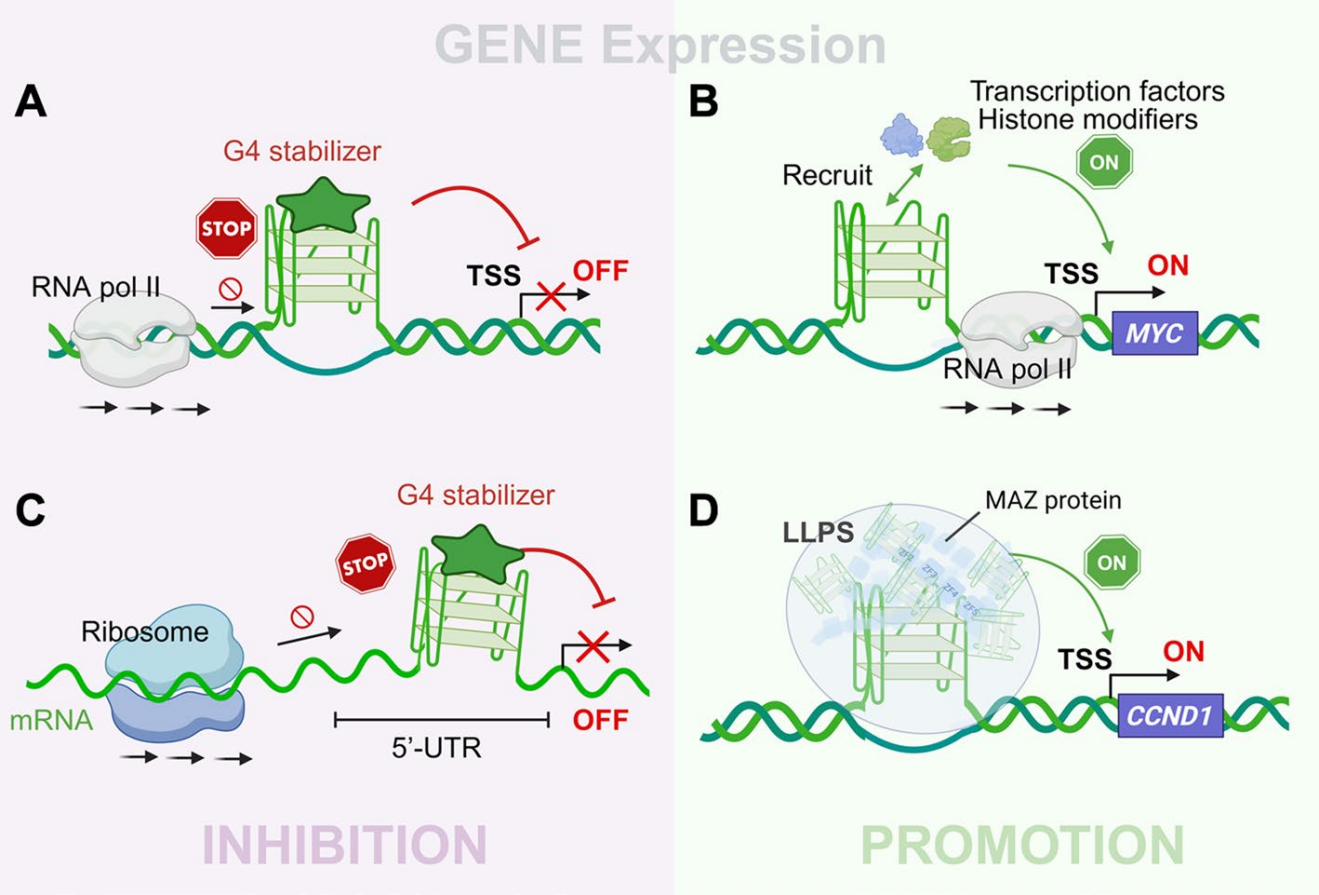
Roles of G4s in gene expression. **A** G4 formation within promoter regions can impede transcription initiation by RNA polymerase. **B** G4 structures in the MYC promoter recruit transcription factors and histone-modifying enzymes, facilitating gene activation. **C** G4 motifs in the 5′ untranslated regions (5′UTRs) of mRNAs modulate translational efficiency and may induce premature transcriptional termination. Three consecutive arrows indicate the direction of sliding. **D** The CCND1-G4 structure serves as a scaffold for MAZ binding, promoting the formation of phase-separated condensates that concentrate transcriptional coactivators and enhance CCND1 transcription. LLPS, liquid–liquid phase separation

Numerous studies have shown that promoter G4 formation influences the expression of key regulatory genes, including *c-MYC* [59], *VEGF* [60, 61], *BCL2* [62, 63], *KRAS* [64, 65], and *KIT* [66, 67]. Early research linked G4 stabilization primarily to transcriptional repression (Fig. 3A). For instance, stabilization of the G4 in the *c-MYC* promoter suppresses its expression [68]. This G4 element in the NHE III1 region modulates transcription via interactions with single-stranded DNA-binding proteins and structure-specific resolving or stabilizing factors, and ligand-mediated stabilization results in *c-MYC* downregulation [69]. Targeting promoter G4s in oncogenes such as *c-MYC* has emerged as a promising anticancer strategy [70, 71]. Similarly, *KRAS* and *SNAIL1* promoters contain G4s that negatively regulate transcription [65, 72, 73]. Recent studies have demonstrated that G4s can also facilitate gene activation. Clustered regularly interspaced short palindromic repeats (CRISPR)-mediated deletion of a G4 in the *MYC* promoter suppresses transcription, indicating that the G4 structure itself recruits transcription factors and chromatin modifiers to enhance transcription, rather than the underlying sequence alone (Fig. 3B) [74]. Other cancer-related gene promoters, such as *OCT4* [75] and *BAP1* [76], show similar G4-mediated activation.

#### G-quadruplex in RNA

Recent advances in detection and structural biology have revealed that RNA G4s (rG4s) are widely present in vivo and play critical roles in post-transcriptional regulation, translational control, and subcellular RNA localization [5]. Thousands of rG4s are predicted across the mammalian transcriptome [77], enriched in mRNAs and noncoding RNAs (ncRNAs), underscrores their widespread biological significance [78]. rG4s are frequently located near 5′ untranslated region (5′-UTR) start sites, suggesting a role in translation initiation. pG4s have been identified in the 5′-UTRs of ~ 3,000 human genes, where they are implicated in translational repression (Fig. 3C) [79, 80]. The first experimental evidence of G4-mediated translational inhibition came from studies of NRAS mRNA, a proto-oncogene essential for regulating cell proliferation and differentiation [81]. More recently, Jodoin et al. demonstrated that colorectal cancer cells and tissues harbor an rG4 structure within the 5′-UTR of *BAG-1* (BCL-2-associated athanogene-1), whose presence significantly reduces the expression of endogenous BAG-1 isoforms [82]. Mechanistically, small nuclear ribonucleoprotein polypeptide A (SNRPA) binds directly to this rG4 element, and SNRPA knockdown in colorectal cancer cells alters BAG-1 protein expression, confirming its regulatory role [83].

Population-scale analyses reveal strong negative selection on central guanines of UTR pG4s, comparable to missense variants in coding regions. At genome-wide association studies (GWAS)-implicated UTR pG4 variant sites, GTEx data indicate significant allelic imbalance across tissues, suggesting that UTR G4 variants contribute to phenotypic diversity [84]. Insulin-like growth factor 2 binding protein 1 (IGF2BP1) regulates *CCN1* mRNA stability via a G4 structure in its 3′-UTR [85].

Long noncoding RNAs (lncRNAs), defined as transcripts exceeding 200 nucleotides, encompass antisense, intronic, divergent, intergenic, and enhancer types, and have attracted increasing attention [86]. Initial evidence for the existence of rG4s in lncRNAs was reported by Jayaraj et al. in 2012 [87]. Subsequent studies have shown that *MALAT1* lncRNA harbors conserved parallel rG4 motifs, which specifically and stably interact with NONO protein both in vitro and in nuclear lysates. Notably, this MALAT1 rG4–NONO interaction can be disrupted in cells by rG4-targeting small molecules, peptides, or l-aptamers [88, 89]. While this review does not cover lncRNA G4s in detail, interested readers are referred to Ref. [90] for a comprehensive discussion.

#### G4-mediated liquid–liquid phase separation

Interestingly, accumulating evidence indicates that G4–mediated transcriptional regulation can involve liquid–liquid phase separation (LLPS). For example, G4s in the *CCND1* (cyclin D1) promoter promote dynamic interactions within phase-separated condensates formed by the MYC-associated zinc finger protein (MAZ), enhancing CCND1 transcription (Fig. 3D) [91].

LLPS is increasingly recognized as a fundamental biophysical mechanism by which cells compartmentalize proteins, DNA, and RNA into membraneless condensates, enabling spatiotemporal regulation of diverse processes, including transcription, RNA metabolism, and DNA repair [92]. At the molecular level, G4 structures exhibit a strong intrinsic ability to drive phase separation. Specifically, G4s can form liquid droplets with histone H1, reducing molecular mobility within droplets, and π–π stacking between quadruplex DNAs may further promote droplet formation. The high LLPS potential of G4s arises from interfaces formed by stacked guanine planes and side surfaces with high charge density [93].

Moreover, abnormally stabilized telomeric G4s in *BRCA2*-deficient cells induce transcription of telomere repeat-containing RNA (TERRA), which promotes the accumulation of telomeric R-loops; these TERRA-containing R-loops in turn trigger LLPS and facilitate telomere synthesis [94]. The Fragile X mental retardation protein (FMRP)-derived RGG peptide has been shown to undergo LLPS with G4-forming Myc-DNA, whereas a point-mutated peptide, in which all arginines were replaced by lysines, failed to phase separate; further truncation experiments indicate that at least five positive charges are required for G4–LLPS [95].

Beyond DNA, RNA G4s can also drive phase separation: an RNA G4 formed within *SHORT ROOT* (*SHR*) mRNA can induce RNA condensates under physiological conditions, suggesting a role in forming phase-separation-like structures in vivo [96]. In minimal LLPS model systems, G4-forming oligonucleotides can co-condense with arginine- and glycine-rich peptides to generate liquid droplets, demonstrating that the G4 structure is critical for mediating RNA–peptide co-condensation [97, 98].

Multimolecular G4s (mG4), formed via interactions between distant genomic loci or across separate nucleic acid strands, can positively regulate transcription. This occurs through the induction of LLPS and selective recruitment of chromatin-remodeling proteins, whereby mG4s establish distal guanine–guanine base-paired DNA networks that generate liquid droplets at DNA loop interfaces to enhance gene expression [99].

#### G-quadruplex in genome instability

**Fig. 4:**
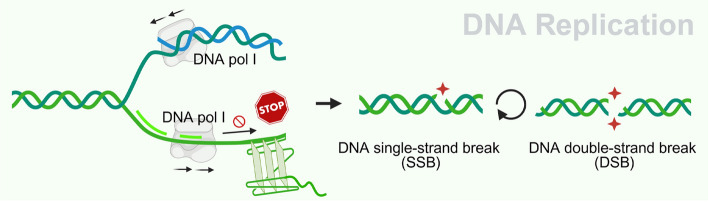
Replication fork stalling and genome instability induced by unresolved G4. Unresolved G4 structures serve as physical impediments to replication fork progression, causing DNA polymerase stalling. This stalling initially leads to the accumulation of single-stranded DNA (ssDNA) gaps, which, if not properly repaired, can be converted into double-strand breaks (DSBs) during subsequent cell divisions. The resulting replication stress promotes the accumulation of DNA lesions—including single-strand breaks (SSBs) and DSBs—ultimately threatening genomic integrity. Two consecutive arrows indicate the direction of sliding

The dynamic formation of G4 structures during DNA replication can induce genomic instability by causing replication fork stalling and DNA breakage (Fig. 4). Helicases act on both DNA and RNA G4s, which are closely linked to the maintenance of genomic and transcriptome stability. In *Saccharomyces cerevisiae*, G4s form in vivo, and the helicase Pif1 plays a critical role in resolving them to prevent replication fork stalling and genomic damage [100].

In human cells, several helicases—including Fanconi anemia complementation group J (FANCJ), Bloom syndrome protein (BLM), and Werner syndrome helicase (WRN)—can unwind G4 structures under physiological conditions [101–103]. FANCJ is a structure-specific DNA helicase that unwinds G4 DNA in vitro with 5′–3′ polarity [104]. G4 structures are particularly enriched during the S phase of the cell cycle [4] and can persist through multiple mitotic divisions without conformational changes. When replication encounters G4 barriers, fork stalling generates single-stranded DNA (ssDNA) gaps, which may be converted into double-strand breaks (DSBs) in daughter cells during subsequent divisions (Fig. 4) [105].

Therefore, both helicase dysfunction and exposure to exogenous G4-stabilizing ligands can impair DNA replication and repair [106]. For instance, in *REV1*-deficient avian DT40 cells, replication fork stalling was mapped to pG4 sites, confirming that stalling results from G4 formation rather than merely G-rich sequences. Moreover, G4-induced replication defects also uncouple DNA synthesis from histone recycling [107]. Loss of FANCJ’s G4-unwinding activity is associated with the accumulation of large genomic deletions near sequences matching G4 motifs [104].

Taken together, G4 structures play crucial roles in telomere biology, transcriptional regulation (particularly of oncogenes), and genome instability (including DNA breakage), providing a solid rationale for the development of small-molecule anticancer therapeutics targeting G4s.

## G-quadruplex: a promising target for anticancer therapy

Given the critical roles of G4s in various biological processes, the development of small molecules that selectively target G4 structures has emerged as a highly promising strategy for cancer therapy. In recent years, research on G4-binding ligands has expanded rapidly, with over 3000 distinct compounds identified that effectively interact with G4s in cellular contexts, underscoring their therapeutic potential [108]. As summarized in Table 1, these ligands have been evaluated across various cancer types. This section focuses on current therapeutic strategies involving G4-targeting small molecules, including both monotherapy and synthetic lethality approaches.

**Table 1:** Overview of the effect of G4 ligands on different cancer types

Ligand	Target G4	Cancer type	Mechanism	Current status	Refs.
RHPS4	Telomere	Melanoma, prostate, non-small cell lung, breast, colon carcinoma	Inducing telomere injury and apoptosis	In vivo	[109]
BRACO19	Telomere	Uterine fibroids, gliomas, osteosarcomas,	Disrupting the integrity of the telomere structure	In vivo	[110]
Schizocommunin derivative (compound 16)	Telomere	Cervical squamous cancer	Inducing cell cycle arrest and apoptosis	In vivo	[111]
Pyridostatin (PDS)	Telomere	Fibrosarcoma	Triggering telomeric DNA damage	In vivo	[112]
TMPyP4	Telomere; c-MYC; hTERT; KRAS	Breast cancer prostate cancer pancreatic cancer	Inhibiting telomerase activity; Decrease in the expression of c-MYC, hTERT, and KRAS	In vivo	[113–115]
Tetra-Pt(bpy)	Telomere	Cervical cancer	Inhibiting telomerase to maintain telomere length; causing telomere DNA damage	In vivo	[116]
QN-302	MDM2	Liposarcoma pancreatic cancer colorectal cancer	Inhibiting MDM2 expression and restoring p53 levels	In vivo Phase I	[117, 118]
Tribenzophenazine analog (MBD)	KRAS	Non-small cell lung cancer	Decreasing KRAS expression	In vivo	[119]
SF3 and TP2 SF3 TP2	mtDNA G4	Triple-negative breast cancer	Disrupting mitochondrial function	In vivo	[120, 121]
A6	mtDNA G4	Breast cancer	Causing mtDNA damage, stimulating cGAS–STING pathway	In vivo	[122]
TA-1	Multiple DNA G4	Liver cancer	Triggering replication-stress-dependent DNA damage, promote 53BP1 expression	In vivo	[123]
CX-5461	Telomere	*BRCA1/2*-deficient tumors	Blocks replication forks and induces ssDNA gaps or breaks	Phase I/II	[124]
a6	Dual-targeting HDAC and G4	Breast cancer	Inducing DNA damage and gene expression changes	In vivo	[125]
Scriptaid	Ribosomal DNA G4	Colorectal cancer	Causing DNA damage	In vivo	[126]

### Targeting telomeric DNA G-quadruplex

Therapeutic strategies aimed at telomeric DNA G4s were initially inspired by the hypothesis that small molecules capable of stabilizing telomeric G4s could physically impede telomerase activity, thereby limiting the replicative potential of cancer cells [127]. In addition, G4 formation can disrupt the T-loop structure, leading to extensive telomeric DNA damage and promoting tumor cell death [128]. These mechanistic insights underscore the importance of rational design of G4-stabilizing compounds to fully exploit the therapeutic potential of G4 biology.

The first evidence supporting telomerase inhibition through G4 stabilization was reported in 1997, when an anthraquinone-based G4 ligand was shown to suppress telomerase activity in a telomeric repeat amplification protocol (TRAP) assay [129]. Since then, a variety of G4-stabilizing molecules have been developed. For instance, RHPS4 induces rapid and potent telomeric DNA damage responses in human transformed fibroblasts and melanoma cells, and in vivo, it inhibits tumor growth in xenograft models derived from various human cancers by promoting telomere dysfunction and apoptosis [109]. BRACO19 selectively binds telomeric G4s, disrupts telomere structural integrity, and induces cellular senescence [110]. The fungal alkaloid schizocommunin has served as a scaffold for designing telomeric G4 ligands, with derivatives exhibiting high affinity for telomeric G4s, promoting their formation and stabilization, inducing DNA damage, disrupting telomere-binding proteins (TRF2 and POT1), causing telomere uncapping, and generating anaphase bridges. In cervical cancer mouse models, these compounds effectively suppressed tumor growth with minimal toxicity [111]. Pyridostatin (PDS) induces and stabilizes telomeric G4s, blocking the protective telomere protein POT1, thereby destabilizing telomeres, triggering extensive DNA damage, and leading to cancer cell death [112]. TMPyP4, a cationic porphyrin considered a nonselective G4 binder, stabilizes telomeric G4s via end-stacking interactions and specifically inhibits telomerase activity in breast cancer cells, although its cellular effects are not entirely G4-dependent [69, 113].

Beyond organic small molecules, metal-based G4 ligands have gained attention due to their diverse geometries, tunable electrochemical properties, and ease of synthesis [8, 130]. For example, Tetra-Pt(bpy) selectively induces telomeric G4 formation while simultaneously inhibiting both telomerase and the alternative lengthening of telomeres (ALT) pathway, rapidly inducing severe telomeric DNA damage and promoting cancer cell death [116, 131].

### Targeting gene expression

An alternative anticancer strategy is to inhibit oncogene expression by stabilizing G4 structures in promoter regions or mRNA 5′-UTRs using small-molecule ligands. TMPyP4 can bind and stabilize G4s in human telomeres and the *c-MYC* promoter, inhibiting telomerase activity and downregulating c-MYC expression (114). Although extensive research has demonstrated that TMPyP4 inhibits *c-MYC* transcription, its lack of G4 selectivity remains an established fact. Other G4 ligands, including QN-1 [132], IZCZ-3 [133], and the thiazole-based peptide TH3 [134], similarly suppress *c-MYC* transcription. Several other oncogenes, such as *BCL-2* [135, 136], *c-KIT* [67], *VEGF* [137], and *hTERT* [138], contain promoter G4s and are susceptible to repression by G4-binding ligands. Beyond classical oncogenes, genes harboring inducible G4s, such as P2 promoter of *mouse double minute 2* (*MDM2*), can also be targeted. For example, QN-302 stabilizes G4s in the *MDM2* P2 promoter, blocking RNA polymerase progression from the P1 promoter, leading to p53 accumulation via the p53–MDM2 autoregulatory loop and triggering apoptosis [117].

The principle of targeting mRNA 5′-UTR G4s mirrors that of DNA promoter G4s, with the main difference being that mRNA targeting disrupts ribosome assembly rather than transcription initiation [139]. rG4s are thermodynamically more stable than DNA G4s [79, 140]. TMPyP4-C14 binds rG4s in the KRAS 5′-UTR, downregulating KRAS protein expression and arresting pancreatic cancer cell growth [115]. Similarly, compounds such as 4,11-bis[2-aminoethylamino]anthra[2,3-*b*]furan-5,10-dione, 4,11-bis(2-aminoethylamino)anthra[2,3-*b*]thiophene-5,10-dione [141], and coumarin-quinolinium derivatives [142] stabilize KRAS rG4s and inhibit translation. The tribenzophenazine analog MBD induces rG4 formation in KRAS G12S-mutant A549/DDP NSCLC cells, downregulates KRAS, suppresses downstream signaling, and inhibits tumor growth in vivo [119]. NRAS expression can be controlled via photo-irradiation with anionic phthalocyanine ZnAPC, which binds NRAS 5′-UTR G4 and induces selective cleavage upon light exposure, reducing NRAS expression and cell viability [143]. PhenDC3, a benchmark G4 ligand, enhances RBM25 binding to the GQ-2 rG4 of BCL-x pre-mRNA, promoting the pro-apoptotic Bcl-xS isoform and inducing apoptosis [144].

### Targeting genome instability and synthetic lethality

Aberrant formation of G4s at inappropriate genomic loci or cell cycle stages can disrupt DNA replication and induce genomic instability by generating DNA damage [145]. Consistently, G4-stabilizing ligands have been shown to stall replication forks and trigger DSBs. For example, PDS induces replication- and transcription-dependent DNA damage in human cancer cells and promotes the formation of nuclear DNA:RNA hybrid structures, further contributing to genomic instability [38, 146]. TA-1, a derivative of tanshinone IIA, stabilizes G4 DNA, induces replication stress-dependent damage. It also enhances 53BP1 expression, activating toxic nonhomologous end joining (NHEJ) repair and apoptosis via the ATM–Chk2–p53 pathway in hepatocellular carcinoma cells [147]. These findings underscore the potential of G4-targeting ligands for synergistic cancer therapies.

Synthetic lethality is a genetic phenomenon in which simultaneous inactivation of two functionally related genes leads to cell death, while loss of either gene alone is nonlethal. Pharmacologically, this can be mimicked using small molecules to inhibit complementary pathways [148, 149]. A canonical example is poly(ADP-ribose) polymerase 1 (PARP1), a key DNA repair enzyme. *BRCA1/2*-deficient cells, impaired in homologous recombination (HR), are highly sensitive to PARP inhibition. PARP inhibitors, including olaparib, talazoparib, niraparib, rucaparib, and veliparib, have demonstrated clinical efficacy, with olaparib approved by the FDA for treating *BRCA*-deficient tumors [150–152].

In 2008, Soldatenkov and colleagues first demonstrated that PARP1 binds intramolecular DNA G4 structures in vitro and becomes catalytically activated upon interaction with G4s located in gene promoters and human telomeres [153]. Subsequent studies showed that PARP1 selectively interacts with promoter-associated G4s, such as those upstream of the KRAS transcription start site, thereby modulating gene expression (72). Beyond transcriptional regulation, PARP1–G4 interactions also play an important role in telomere biology. In BJ-hTERT fibroblasts, PARP1 is recruited to telomeric G4 DNA stabilized by the telomere-targeting ligand RHPS4, resulting in PARP1 activation [154]. More recent work has revealed that G4-forming sequences within the *c-KIT* promoter stimulate PARP1 enzymatic activity, indicating that G4 structural features can modulate PARP1 function [155]. Notably, a G4-forming sequence has also been identified in the promoter of the PARP1 gene itself, adopting a unique intramolecular (3 + 1) hybrid G-quadruplex topology[25, 156]. These findings suggest that stabilization of promoter-associated G4s may represent a promising therapeutic strategy, particularly in *BRCA*-deficient cancers.

**Fig. 5:**
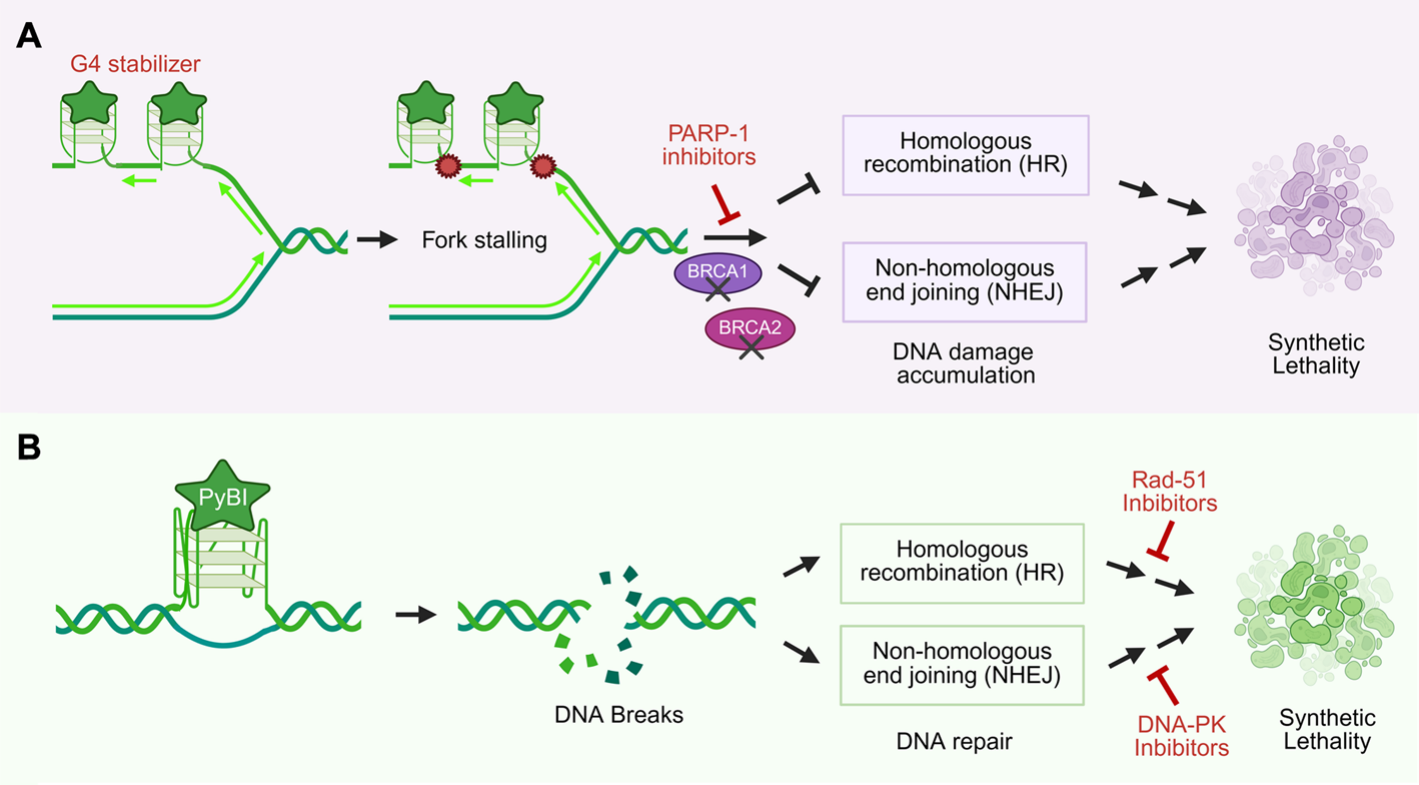
Mechanisms of DNA damage and synthetic lethality induced by G4 stabilizers. **A** G4 stabilizers stall replication forks, inducing DNA gaps or breaks, which are repaired by *BRCA*-dependent homologous recombination (HR) and nonhomologous end joining (NHEJ) pathways. In the context of *BRCA* deficiency or combined PARP inhibition, repair is impaired, leading to synthetic lethality. **B** PyBI selectively binds parallel/heterogeneous G4 DNA, causing replication fork stalling and DNA breaks while activating HR and NHEJ pathways. Under combined inhibition of Rad51 and DNA-PK, PyBI induces synthetic lethality

Consistent with this concept, the G4-stabilizing ligand CX-5461 exhibits selective cytotoxicity toward *BRCA*-deficient cancer cells and patient-derived xenograft (PDX) models, including tumors resistant to PARP inhibitors. Mechanistically, CX-5461 and its analog CX-3543 induce replication fork stalling and DNA damage that require BRCA- and NHEJ-dependent repair, leading to synthetic lethality when repair fails [124] (Fig. 5A). CX-5461 is currently undergoing phase I/II clinical trials for *BRCA1/2*-deficient tumors [157]. In addition, other G4 ligands, such as QN-302 and PDS, have shown antitumor activity and enhanced telomere instability in *BRCA*-deficient contexts [158]. Synergistic tumor suppression has also been observed when PARP1 inhibition is combined with telomere-targeting G4 ligands, highlighting the therapeutic potential of co-targeting PARP–G4 pathways [154]. A thieno[3,2-*c*]quinolin-4(5*H*)-one-based dual G4/PARP ligand stabilizes G4s, suppresses PARylation, and induces telomere-associated DNA damage, thereby inhibiting proliferation of *BRCA2*-deficient tumor cells [159]. Beyond PARP1, other members of the PARP family, such as PARP3, also regulate G4 stability. Chemical stabilization of G4 structures in PARP3-deficient cells induces widespread DSBs and results in synthetic lethality [160]. Although research on PARP–G4 interactions is still limited, the crucial roles of both PARP proteins and G4 structures in DNA repair and cancer therapy highlight the significant potential of PARP–G4-targeted strategies.

In addition to combination therapies involving PARP1 inhibition, G4 stabilizers also exhibit synergistic lethality with other small-molecule agents. For instance, PDS acts synergistically with the DNA-PK inhibitor NU7441 or with *BRCA2*-deficient cells, enhancing the cytotoxicity in cells with compromised DSB repair [148]. Co-treatment with the EZH2 inhibitor EPZ-6438 and PDS selectively kills *BRCA2*-deficient alternative lengthening of telomeres (ALT)-like cancer cells, a mechanism involving telomeric G4 stabilization and TERRA-R-loop-induced liquid–liquid phase separation [94]. Pyridine-bis(benzimidazole) (PyBI), a selective stabilizer of parallel and hybrid G4 conformations, increases G4 formation, triggers DNA damage, and activates both HR and NHEJ repair pathways; consequently, it exhibits synthetic lethality when combined with HR or NHEJ inhibitors [161] (Fig. 5B). Loss of UBE2N or RNF168 further enhances the cytotoxicity of CX-5461 and PDS [162]. CX-5461 also induces p53-independent apoptosis in α-thalassemia/mental retardation X-linked (ATRX)-deficient glioma stem cells and achieves selective lethality in vivo when combined with ionizing radiation [163]. Additionally, G4-binding molecule CM03 and the histone deacetylase (HDAC) inhibitor SAHA (suberanilohydroxamic acid) exhibit synergistic cytotoxicity in pancreatic cancer cells [164]. Interestingly, HDAC inhibitor-induced chromatin relaxation can further promote G4 formation in promoter regions, expanding the potential for targeted G4-based therapies [56]. Huang and colleagues designed dual G4/HDAC-targeting compounds, among which compound a6 markedly inhibits proliferation in multiple TNBC cell lines and MDA-MB-231 xenografts [125]. The HDAC inhibitor Scriptaid binds ribosomal DNA G4s, inducing DNA damage and cytotoxicity [165].

Moreover, targeted G4 strategies show promise in immunotherapy. For example, TMPyP4 inhibits cancer cell proliferation and induces G2/M arrest, while activating the cGAS–STING pathway through DNA damage, thereby promoting CD8^+^ T-cell activation and dendritic cell maturation [166]. Capranico and colleagues reported that G4 ligands can trigger the cGAS–STING pathway, leading to type I interferon and ISG signaling [167, 168]. The extent of immune gene activation depends on ligand affinity for G4s, while excessive cytotoxicity reduces this effect [169]. Combined treatment with TMPyP4 and anti-PD1 antibodies exhibits synergistic antitumor effects in colorectal cancer [166]. Drug administration sequence is also critical. For instance, RHPS4 shows strong synergy with camptothecins in a sequence-dependent manner. In mice, irinotecan followed by RHPS4 effectively suppresses tumor growth and improves survival [170].

### Targeting mitochondrial G-quadruplex

G4 structures can form not only in the nucleus but also in mitochondria, which also contain double-stranded DNA. However, the study of mitochondrial G4s has been limited by the lack of effective detection tools. Currently, two main strategies are employed: synthetic antibodies and fluorescent probes. Synthetic antibodies, such as BG4, allow visualization of G4 structures in living cells [171]. To detect G4s specifically in mtDNA, Sjoerd Wanrooij and colleagues engineered a cell model in which BG4 is selectively targeted to mitochondria. Using the mtG4-ChIP protocol, they showed that replication stalling in mtDNA promotes G4 formation, which impairs replication fork progression and leads to mtDNA depletion [172]. Alternatively, fluorescent probes have been used to directly visualize mitochondrial G4s [173–175].

Targeting mitochondrial G4s has recently emerged as a promising anticancer strategy. For example, the fluorescent G4 ligand 3,6-bis(1-methyl-4-vinylpyridinium) carbazole diiodide (BMVC) selectively relocates from the nucleus to mitochondria in cancer cells. This shift confirming mtDNA G4 formation and leads to suppression mitochondrial gene expression [120, 121, 176]. Similarly, a triphenylamine-based analog, A6, induces extensive mtDNA damage, activating the cGAS–STING pathway, promoting cytokine production and dendritic cell (DC) maturation, and significantly suppressing tumor growth and metastasis in 4T1 tumor-bearing mice by remodeling the tumor microenvironment (TME) [122]. Additionally, the FDA-approved drug pyrvinium pamoate (PP) selectively binds mitochondrial G4s in pancreatic ductal adenocarcinoma (PDAC) cells, reducing mitochondrial RNA expression by ~ 90% and exhibiting potent anticancer activity [177].

The distinction between nuclear and mitochondrial G4 targeting is fundamental. Nuclear G4 targeting elicits complex, often delayed responses involving genomic stress and immune signaling, whereas mitochondrial G4 targeting exerts a direct metabolic assault leading to apoptosis. Rational drug design will benefit from developing organelle-selective G4 ligands or combination strategies that exploit both pathways, providing a multipronged approach to disrupt cancer cell survival.

## Challenges and future directions of G4-targeted therapeutics

### Translational gap and pharmacological limitations

Despite the large number of G4 ligands reported in the literature [127, 157, 178, 179], only a few have advanced to preclinical evaluation, and even fewer have reached clinical trials. Notable examples include CX-5461 [163, 180] and QN-302 [118, 178], while many other candidates have not advanced further owing to limited efficacy or toxicity issues. These observations highlight a substantial translational gap between in vitro activity and in vivo therapeutic efficacy for G4-targeted agents.

Pharmacokinetic and pharmacodynamic limitations are major contributors to this gap. Many planar aromatic ligands exhibit poor solubility, limited membrane permeability, and a tendency to self-aggregate, resulting in reduced bioavailability and cellular uptake [181, 182]. In addition, most ligands display limited selectivity, often interacting with multiple G4 topologies or even duplex DNA [127, 179]. The complex intracellular environment—including protein–G4 interactions, epigenetic modifications, single nucleotide polymorphisms, and telomere-associated proteins—can further modulate ligand performance [4, 171].

### Topology selectivity and off-target effects

A central challenge in G4 drug development is discriminating among different G4 topologies [19, 183]. G4 structures are highly polymorphic, adopting parallel, antiparallel, or hybrid conformations [19, 57]. Most existing ligands bind multiple topologies nonspecifically, potentially stabilizing off-target G4s and causing unintended transcriptional or translational effects [114, 127, 184]. For instance, ligands targeting the *MYC* promoter may inadvertently affect the expression of other genes, complicating the interpretation of biological outcomes [114, 184].

Moreover, research on G4 destabilizers remains limited, yet these agents also hold potential value. In certain contexts, destabilizing specific G4 structures can either upregulate or downregulate gene expression, providing an alternative means of modulating biological processes. Although studies are scarce, further exploration of G4 destabilization strategies could expand the therapeutic repertoire of G4-targeted approaches.

### Design principles for next-generation G4 ligands

To enhance selectivity and translational potential, next-generation G4 ligands should be rationally designed by integrating chemical, structural, and biological considerations. First, topology and sequence selectivity is essential. Ligands should preferentially recognize specific G4 topologies or sequence contexts to minimize off-target effects. High-resolution structural information obtained from nuclear magnetic resonance (NMR) or X-ray [36] crystallography can guide the optimization of aromatic cores, stacking surfaces, and groove- or loop-interacting substituents [183]. Second, optimization of physicochemical properties is critical for in vivo application. Solubility, membrane permeability, chemical stability, and enzymatic resistance should be optimized, following guidelines such as Lipinski’s Rule of Five to overcome pharmacokinetic limitations [181, 182]. Third, functional differentiation between probes and therapeutics should be emphasized. While some ligands are valuable as chemical probes that promote or stabilize nonphysiological G4 structures, such chaperone-like activities may limit their therapeutic utility and should be carefully evaluated in drug development pipelines [185]. Finally, cellular context must be considered. G4 formation and stability are modulated by protein binding, epigenetic state, and telomere-associated factors. Incorporating these contextual determinants into ligand design will be essential for predicting biological outcomes and improving therapeutic precision [183, 185].

### Emerging strategies and preclinical development

To overcome the limitations in selectivity and clinical translation discussed above, several emerging strategies are being actively explored in the preclinical development of G4-targeted therapeutics. Combination approaches represent a particularly promising avenue, including the exploitation of synthetic lethality—such as the application of CX-5461 in *BRCA1/2*-deficient tumors—as well as the integration of G4 ligands with conventional chemotherapy or immunotherapy to enhance efficacy while mitigating systemic toxicity [124, 157]. In parallel, immunomodulatory effects of G4 ligands are gaining attention, as low-dose G4 stabilization has been shown to activate innate immune signaling and autophagy in cancer cells, thereby providing potential synergy with immunotherapeutic interventions [167, 168]. Advances in preclinical evaluation platforms, including three-dimensional culture systems, organoids, and patient-derived xenograft models, offer more physiologically relevant contexts to assess therapeutic responses. Moreover, improving ligand stability and pharmacokinetic properties remains critical for successful in vivo application. The development of fluorescent derivatives and cellular probes has further facilitated real-time monitoring of G4 dynamics, enabling both mechanistic studies and efficacy assessment [39, 40]. Finally, targeted delivery strategies—such as conjugation to oligonucleotides [186, 187], peptide nucleic acids [188], or dCas9-based systems [189, 190]—are emerging as powerful approaches to achieve locus-specific G4 modulation, thereby improving selectivity and reducing off-target effects.

### Summary and outlook

Next-generation G4-targeted therapeutics should prioritize enhanced selectivity, improved translational efficiency, and minimization of off-target effects. By leveraging high-resolution structural information, rational chemical design, advanced delivery strategies, and physiologically relevant preclinical models, it is feasible to develop G4 ligands with stronger specificity, optimized pharmacokinetics, and more reliable therapeutic efficacy. Additionally, further exploration of G4 destabilizers could provide complementary approaches for modulating gene expression and expanding the therapeutic scope of G4-targeted interventions. These efforts collectively promise new avenues for cancer treatment and other disease applications.

## Data Availability

No datasets were generated or analyzed during the current study.

## Abbreviations

5′-UTRs: 5′-untranslated regions
ALT: Alternative lengthening of telomeres
ATRX: α-Thalassemia/mental retardation X-linked
BAG-1: BCL-2-associated athanogene-1
BLM: Bloom syndrome protein
CCND1: Cyclin D1
ChIP: Chromatin immunoprecipitation
DMS: Dimethyl sulfate
DPAT: Diphenylaminothiophene
DSBs: DNA double-strand breaks
EMSAs: Electrophoretic mobility shift assays
EMT: Epithelial–mesenchymal transition
FANCJ: Fanconi anemia complementation group J
FDA: Food and Drug Administration
G4s: G-quadruplexes
GMP: Guanosine monophosphate
GSCs: Glioma stem cells
HDAC: Histone deacetylase
HR: Homologous recombination
IR: Ionizing radiation
LLPS: Liquid–liquid phase separation
lncRNAs: Long non-coding RNAs (lncRNAs)
MAZ: MYC-associated zinc finger protein
MDM2: Mouse double minute 2
mG4: Multimolecular G4s
mtDNA: Mitochondrial DNA
NHEJ: Non-homologous end joining
NMR: Nuclear magnetic resonance
PARP1: Poly(ADP-ribose) polymerase 1
PDAC: Pancreatic ductal adenocarcinoma
PDS: Pyridostatin
pG4s or PQSs: Potential G4-forming sequences
PP: Pyrvinium Pamoate
PyBI: Pyridine-bis(benzimidazole)
SNRPA: Small nuclear ribonucleoprotein polypeptide A
ssDNA: Single-stranded DNA
TERRA: Telomeric repeat-containing RNA
TME: Tumor microenvironment
TNBC: Triple-negative breast cancer
TPATP: Triphenylamine-thiophene
TRAP: Telomeric repeat amplification protocol
WRN: Werner syndrome helicase

## References

[1] Leontis NB, Stombaugh J, Westhof E. The non-Watson-Crick base pairs and their associated isostericity matrices. Nucleic Acids Res. 2002;30:3497–531. 12177293 10.1093/nar/gkf481 PMC134247

[2] Rhodes D, Lipps HJ. G-quadruplexes and their regulatory roles in biology. Nucleic Acids Res. 2015;43:8627–37. 26350216 10.1093/nar/gkv862 PMC4605312

[3] Johnson SA, et al. BG4 antibody can recognize telomeric G-quadruplexes harboring destabilizing base modifications and lesions. Nucleic Acids Res. 2024;52:1763–78. 38153143 10.1093/nar/gkad1209 PMC10939409

[4] Di Antonio M, et al. Single-molecule visualization of DNA G-quadruplex formation in live cells. Nat Chem. 2020;12:832–7. 32690897 10.1038/s41557-020-0506-4 PMC7610488

[5] Varshney D, Spiegel J, Zyner K, Tannahill D, Balasubramanian S. The regulation and functions of DNA and RNA G-quadruplexes. Nat Rev Mol Cell Biol. 2020;21:459–74. 32313204 10.1038/s41580-020-0236-x PMC7115845

[6] Kosiol N, Juranek S, Brossart P, Heine A, Paeschke K. G-quadruplexes: a promising target for cancer therapy. Mol Cancer. 2021;20:40. 33632214 10.1186/s12943-021-01328-4 PMC7905668

[7] Awadasseid A, Ma X, Wu Y, Zhang W. G-quadruplex stabilization via small-molecules as a potential anti-cancer strategy. Biomed Pharmacother. 2021;139:111550. 33831835 10.1016/j.biopha.2021.111550

[8] Asamitsu S, Bando T, Sugiyama H. Ligand design to acquire specificity to intended G-quadruplex structures. Chemistry. 2019;25:417–30. 30051593 10.1002/chem.201802691

[9] Gellert M, Lipsett MN, Davies DR. Helix formation by guanylic acid. Proc Natl Acad Sci U S A. 1962;48:2013–8. 13947099 10.1073/pnas.48.12.2013 PMC221115

[10] Guschlbauer W, Chantot JF, Thiele D. Four-stranded nucleic acid structures 25 years later: from guanosine gels to telomer DNA. J Biomol Struct Dyn. 1990;8:491–511. 2100515 10.1080/07391102.1990.10507825

[11] Pochon F, Michelson AM. Polynucleotides. VI. Interaction between polyguanylic acid and polycytidylic acid. Proc Natl Acad Sci U S A. 1965;53:1425–30. 5217645 10.1073/pnas.53.6.1425 PMC219873

[12] Cadoni E, De Paepe L, Manicardi A, Madder A. Beyond small molecules: targeting G-quadruplex structures with oligonucleotides and their analogues. Nucleic Acids Res. 2021;49:6638–59. 33978760 10.1093/nar/gkab334 PMC8266634

[13] Sato K, Knipscheer P. G-quadruplex resolution: from molecular mechanisms to physiological relevance. DNA Repair (Amst). 2023;130:103552. 37572578 10.1016/j.dnarep.2023.103552

[14] Oyaghire SN, et al. RNA G-quadruplex invasion and translation inhibition by antisense γ-peptide nucleic acid oligomers. Biochemistry. 2016;55:1977–88. 26959335 10.1021/acs.biochem.6b00055

[15] Zimmerman SB, Cohen GH, Davies DR. X-ray fiber diffraction and model-building study of polyguanylic acid and polyinosinic acid. J Mol Biol. 1975;92:181–92. 1142423 10.1016/0022-2836(75)90222-3

[16] Sen D, Gilbert W. A sodium-potassium switch in the formation of four-stranded G4-DNA. Nature. 1990;344:410–4. 2320109 10.1038/344410a0

[17] Bochman ML, Paeschke K, Zakian VA. DNA secondary structures: stability and function of G-quadruplex structures. Nat Rev Genet. 2012;13:770–80. 23032257 10.1038/nrg3296 PMC3725559

[18] Hud NV, Smith FW, Anet FA, Feigon J. The selectivity for K+ versus Na+ in DNA quadruplexes is dominated by relative free energies of hydration: a thermodynamic analysis by ^1^H NMR. Biochemistry. 1996;35:15383–90. 8952490 10.1021/bi9620565

[19] Burge S, Parkinson GN, Hazel P, Todd AK, Neidle S. Quadruplex DNA: sequence, topology and structure. Nucleic Acids Res. 2006;34:5402–15. 17012276 10.1093/nar/gkl655 PMC1636468

[20] Spiegel J, Adhikari S, Balasubramanian S. The structure and function of DNA G-quadruplexes. Trends Chem. 2020;2:123–36. 32923997 10.1016/j.trechm.2019.07.002 PMC7472594

[21] Esposito V, et al. A topological classification of G-quadruplex structures. Nucleosides Nucleotides Nucleic Acids. 2007;26:1155–9. 18058556 10.1080/15257770701527059

[22] Parkinson GN, Lee MP, Neidle S. Crystal structure of parallel quadruplexes from human telomeric DNA. Nature. 2002;417:876–80. 12050675 10.1038/nature755

[23] Guédin A, Gros J, Alberti P, Mergny JL. How long is too long? Effects of loop size on G-quadruplex stability. Nucleic Acids Res. 2010;38:7858–68. 20660477 10.1093/nar/gkq639 PMC2995061

[24] Huppert JL, Balasubramanian S. Prevalence of quadruplexes in the human genome. Nucleic Acids Res. 2005;33:2908–16. 15914667 10.1093/nar/gki609 PMC1140081

[25] Chambers VS, et al. High-throughput sequencing of DNA G-quadruplex structures in the human genome. Nat Biotechnol. 2015;33:877–81. 26192317 10.1038/nbt.3295

[26] Marsico G, et al. Whole genome experimental maps of DNA G-quadruplexes in multiple species. Nucleic Acids Res. 2019;47:3862–74. 30892612 10.1093/nar/gkz179 PMC6486626

[27] Kim N. The interplay between G-quadruplex and transcription. Curr Med Chem. 2019;26:2898–917. 29284393 10.2174/0929867325666171229132619 PMC6026074

[28] Prorok P, et al. Involvement of G-quadruplex regions in mammalian replication origin activity. Nat Commun. 2019;10:3274. 31332171 10.1038/s41467-019-11104-0 PMC6646384

[29] Li F, Zhou J. G-quadruplexes from non-coding RNAs. J Mol Med (Berl). 2023;101:621–35. 37069370 10.1007/s00109-023-02314-7

[30] Lee CY, Joshi M, Wang A, Myong S. 5’UTR G-quadruplex structure enhances translation in size dependent manner. Nat Commun. 2024;15:3963. 38729943 10.1038/s41467-024-48247-8 PMC11087576

[31] Varshney D, et al. RNA G-quadruplex structures control ribosomal protein production. Sci Rep. 2021;11:22735. 34815422 10.1038/s41598-021-01847-6 PMC8611094

[32] Datta A, Pollock KJ, Kormuth KA, Brosh RM Jr. G-Quadruplex assembly by ribosomal DNA: emerging roles in disease pathogenesis and cancer biology. Cytogenet Genome Res. 2021;161:285–96. 34469893 10.1159/000516394 PMC8455414

[33] Dahal S, Siddiqua H, Katapadi VK, Iyer D, Raghavan SC. Characterization of G4 DNA formation in mitochondrial DNA and their potential role in mitochondrial genome instability. FEBS J. 2022;289:163–82. 34228888 10.1111/febs.16113

[34] Sahayasheela VJ, Yu Z, Hidaka T, Pandian GN, Sugiyama H. Mitochondria and G-quadruplex evolution: an intertwined relationship. Trends Genet. 2023;39:15–30. 36414480 10.1016/j.tig.2022.10.006 PMC9772288

[35] Zhang X, et al. G-quadruplex structures at the promoter of HOXC10 regulate its expression. Biochim Biophys Acta Gene Regul Mech. 2018;1861:1018–28. 30343692 10.1016/j.bbagrm.2018.09.004

[36] Adrian M, Heddi B, Phan AT. NMR spectroscopy of G-quadruplexes. Methods (San Diego, Calif). 2012;57:11–24. 22633887 10.1016/j.ymeth.2012.05.003

[37] Henderson A, et al. Detection of G-quadruplex DNA in mammalian cells. Nucleic Acids Res. 2017;45:6252. 28449109 10.1093/nar/gkx300 PMC5449604

[38] Rodriguez R, et al. Small-molecule-induced DNA damage identifies alternative DNA structures in human genes. Nat Chem Biol. 2012;8:301–10. 22306580 10.1038/nchembio.780 PMC3433707

[39] Shivalingam A, et al. The interactions between a small molecule and G-quadruplexes are visualized by fluorescence lifetime imaging microscopy. Nat Commun. 2015;6:8178. 26350962 10.1038/ncomms9178 PMC4579598

[40] Zhang S, et al. Real-time monitoring of DNA G-quadruplexes in living cells with a small-molecule fluorescent probe. Nucleic Acids Res. 2018;46:7522–32. 30085206 10.1093/nar/gky665 PMC6125622

[41] Hänsel-Hertsch R, Spiegel J, Marsico G, Tannahill D, Balasubramanian S. Genome-wide mapping of endogenous G-quadruplex DNA structures by chromatin immunoprecipitation and high-throughput sequencing. Nat Protoc. 2018;13:551–64. 29470465 10.1038/nprot.2017.150

[42] Lyu J, Shao R, Kwong Yung PY, Elsässer SJ. Genome-wide mapping of G-quadruplex structures with CUT&Tag. Nucleic Acids Res. 2022;50:e13. 34792172 10.1093/nar/gkab1073 PMC8860588

[43] Zheng KW, et al. Detection of genomic G-quadruplexes in living cells using a small artificial protein. Nucleic Acids Res. 2020;48:11706–20. 33045726 10.1093/nar/gkaa841 PMC7672459

[44] Shay JW, Wright WE. Telomeres and telomerase: three decades of progress. Nat Rev Genet. 2019;20:299–309. 30760854 10.1038/s41576-019-0099-1

[45] de Lange T. Shelterin: the protein complex that shapes and safeguards human telomeres. Genes Dev. 2005;19:2100–10. 16166375 10.1101/gad.1346005

[46] Monsen RC, Chakravarthy S, Dean WL, Chaires JB, Trent JO. The solution structures of higher-order human telomere G-quadruplex multimers. Nucleic Acids Res. 2021;49:1749–68. 33469644 10.1093/nar/gkaa1285 PMC7897503

[47] Shay JW. Role of telomeres and telomerase in aging and cancer. Cancer Discov. 2016;6:584–93. 27029895 10.1158/2159-8290.CD-16-0062 PMC4893918

[48] Gao J, Pickett HA. Targeting telomeres: advances in telomere maintenance mechanism-specific cancer therapies. Nat Rev Cancer. 2022;22:515–32. 35790854 10.1038/s41568-022-00490-1

[49] Neidle S, Parkinson G. Telomere maintenance as a target for anticancer drug discovery. Nat Rev Drug Discov. 2002;1:383–93. 12120414 10.1038/nrd793

[50] Moyzis RK, et al. A highly conserved repetitive DNA sequence, (TTAGGG)n, present at the telomeres of human chromosomes. Proc Natl Acad Sci U S A. 1988;85:6622–6. 3413114 10.1073/pnas.85.18.6622 PMC282029

[51] Sfeir AJ, Chai W, Shay JW, Wright WE. Telomere-end processing the terminal nucleotides of human chromosomes. Mol Cell. 2005;18:131–8. 15808515 10.1016/j.molcel.2005.02.035

[52] Sundquist WI, Klug A. Telomeric DNA dimerizes by formation of guanine tetrads between hairpin loops. Nature. 1989;342:825–9. 2601741 10.1038/342825a0

[53] Williamson JR, Raghuraman MK, Cech TR. Monovalent cation-induced structure of telomeric DNA: the G-quartet model. Cell. 1989;59:871–80. 2590943 10.1016/0092-8674(89)90610-7

[54] Tang J, et al. G-quadruplex preferentially forms at the very 3’ end of vertebrate telomeric DNA. Nucleic Acids Res. 2008;36:1200–8. 18158301 10.1093/nar/gkm1137 PMC2275102

[55] Xu Y. Chemistry in human telomere biology: structure, function and targeting of telomere DNA/RNA. Chem Soc Rev. 2011;40:2719–40. 21301727 10.1039/c0cs00134a

[56] Hänsel-Hertsch R, et al. G-quadruplex structures mark human regulatory chromatin. Nat Genet. 2016;48:1267–72. 27618450 10.1038/ng.3662

[57] Huppert JL, Balasubramanian S. G-quadruplexes in promoters throughout the human genome. Nucleic Acids Res. 2007;35:406–13. 17169996 10.1093/nar/gkl1057 PMC1802602

[58] Todd AK, Haider SM, Parkinson GN, Neidle S. Sequence occurrence and structural uniqueness of a G-quadruplex in the human c-kit promoter. Nucleic Acids Res. 2007;35:5799–808. 17720713 10.1093/nar/gkm609 PMC2034477

[59] Simonsson T, Pecinka P, Kubista M. DNA tetraplex formation in the control region of c-myc. Nucleic Acids Res. 1998;26:1167–72. 9469822 10.1093/nar/26.5.1167 PMC147388

[60] Agrawal P, Hatzakis E, Guo K, Carver M, Yang D. Solution structure of the major G-quadruplex formed in the human VEGF promoter in K+: insights into loop interactions of the parallel G-quadruplexes. Nucleic Acids Res. 2013;41:10584–92. 24005038 10.1093/nar/gkt784 PMC3905851

[61] Guo K, Gokhale V, Hurley LH, Sun D. Intramolecularly folded G-quadruplex and i-motif structures in the proximal promoter of the vascular endothelial growth factor gene. Nucleic Acids Res. 2008;36:4598–608. 18614607 10.1093/nar/gkn380 PMC2504309

[62] Dai J, Chen D, Jones RA, Hurley LH, Yang D. NMR solution structure of the major G-quadruplex structure formed in the human BCL2 promoter region. Nucleic Acids Res. 2006;34:5133–44. 16998187 10.1093/nar/gkl610 PMC1636422

[63] Onel B, et al. A new G-quadruplex with hairpin loop immediately upstream of the human BCL2 P1 promoter modulates transcription. J Am Chem Soc. 2016;138:2563–70. 26841249 10.1021/jacs.5b08596 PMC5019542

[64] D’Aria F, Pagano B, Petraccone L, Giancola C. KRAS promoter G-quadruplexes from sequences of different length: a physicochemical study. Int J Mol Sci. 2021. 10.3390/ijms22010448. 33466280 10.3390/ijms22010448 PMC7795837

[65] Cogoi S, Xodo LE. G-quadruplex formation within the promoter of the KRAS proto-oncogene and its effect on transcription. Nucleic Acids Res. 2006;34:2536–49. 16687659 10.1093/nar/gkl286 PMC1459413

[66] Rigo R, Sissi C. Characterization of G4–G4 crosstalk in the c-KIT promoter region. Biochemistry. 2017;56:4309–12. 28763217 10.1021/acs.biochem.7b00660

[67] Zorzan E, et al. Targeting canine KIT promoter by candidate DNA G-quadruplex ligands. J Pharmacol Exp Ther. 2018;367:461–72. 30275152 10.1124/jpet.118.248997

[68] Siddiqui-Jain A, Grand CL, Bearss DJ, Hurley LH. Direct evidence for a G-quadruplex in a promoter region and its targeting with a small molecule to repress c-MYC transcription. Proc Natl Acad Sci U S A. 2002;99:11593–8. 12195017 10.1073/pnas.182256799 PMC129314

[69] Moraca F, et al. Repurposing FDA-approved drugs to target G-quadruplexes in breast cancer. Eur J Med Chem. 2025;285:117245. 39793440 10.1016/j.ejmech.2025.117245

[70] Chen H, Liu H, Qing G. Targeting oncogenic Myc as a strategy for cancer treatment. Signal Transduct Target Ther. 2018;3:5. 29527331 10.1038/s41392-018-0008-7 PMC5837124

[71] Duffy MJ, O’Grady S, Tang M, Crown J. MYC as a target for cancer treatment. Cancer Treat Rev. 2021;94:102154. 33524794 10.1016/j.ctrv.2021.102154

[72] Cogoi S, Paramasivam M, Membrino A, Yokoyama KK, Xodo LE. The KRAS promoter responds to Myc-associated zinc finger and poly(ADP-ribose) polymerase 1 proteins, which recognize a critical quadruplex-forming GA-element. J Biol Chem. 2010;285:22003–16. 20457603 10.1074/jbc.M110.101923 PMC2903372

[73] Wang Y, et al. The SNAIL1 promoter contains G-quadruplex structures regulating its gene expression and DNA replication. Exp Cell Res. 2020;394:112158. 32610184 10.1016/j.yexcr.2020.112158

[74] Esain-Garcia I, et al. G-quadruplex DNA structure is a positive regulator of MYC transcription. Proc Natl Acad Sci USA. 2024;121:e2320240121. 38315865 10.1073/pnas.2320240121 PMC10873556

[75] Renčiuk D, et al. G-quadruplex formation in the Oct4 promoter positively regulates Oct4 expression. Biochim Biophys Acta Gene Regul Mech. 2017;1860:175–83. 27863263 10.1016/j.bbagrm.2016.11.002

[76] Li Y, et al. G-quadruplexes in the BAP1 promoter positively regulate its expression. Exp Cell Res. 2018;369:147–57. 29787736 10.1016/j.yexcr.2018.05.016

[77] Kwok CK, Marsico G, Sahakyan AB, Chambers VS, Balasubramanian S. rG4-seq reveals widespread formation of G-quadruplex structures in the human transcriptome. Nat Methods. 2016;13:841–4. 27571552 10.1038/nmeth.3965

[78] Dumas L, Herviou P, Dassi E, Cammas A, Millevoi S. G-quadruplexes in RNA biology: recent advances and future directions. Trends Biochem Sci. 2021;46:270–83. 33303320 10.1016/j.tibs.2020.11.001

[79] Bugaut A, Balasubramanian S. 5’-UTR RNA G-quadruplexes: translation regulation and targeting. Nucleic Acids Res. 2012;40:4727–41. 22351747 10.1093/nar/gks068 PMC3367173

[80] Beaudoin JD, Perreault JP. Exploring mRNA 3’-UTR G-quadruplexes: evidence of roles in both alternative polyadenylation and mRNA shortening. Nucleic Acids Res. 2013;41:5898–911. 23609544 10.1093/nar/gkt265 PMC3675481

[81] Kumari S, Bugaut A, Huppert JL, Balasubramanian S. An RNA G-quadruplex in the 5’ UTR of the NRAS proto-oncogene modulates translation. Nat Chem Biol. 2007;3:218–21. 17322877 10.1038/nchembio864 PMC2206252

[82] Jodoin R, Carrier JC, Rivard N, Bisaillon M, Perreault JP. G-quadruplex located in the 5’UTR of the BAG-1 mRNA affects both its cap-dependent and cap-independent translation through global secondary structure maintenance. Nucleic Acids Res. 2019;47:10247–66. 31504805 10.1093/nar/gkz777 PMC6821271

[83] Bolduc F, Turcotte MA, Perreault JP. The small nuclear ribonucleoprotein polypeptide A (SNRPA) binds to the G-quadruplex of the BAG-1 5’UTR. Biochimie. 2020;176:122–7. 32629040 10.1016/j.biochi.2020.06.013

[84] Lee DSM, Ghanem LR, Barash Y. Integrative analysis reveals RNA G-quadruplexes in UTRs are selectively constrained and enriched for functional associations. Nat Commun. 2020;11:527. 31988292 10.1038/s41467-020-14404-y PMC6985247

[85] Rana P, Ujjainiya R, Bharti V, Maiti S, Ekka MK. IGF2BP1-mediated regulation of CCN1 expression by specific binding to G-quadruplex structure in its 3’UTR. Biochemistry. 2024;63:2166–82. 39133064 10.1021/acs.biochem.4c00172

[86] Kopp F, Mendell JT. Functional classification and experimental dissection of long noncoding RNAs. Cell. 2018;172:393–407. 29373828 10.1016/j.cell.2018.01.011 PMC5978744

[87] Jayaraj GG, Pandey S, Scaria V, Maiti S. Potential G-quadruplexes in the human long non-coding transcriptome. RNA Biol. 2012;9:81–6. 22258148 10.4161/rna.9.1.18047

[88] Mou X, Liew SW, Kwok CK. Identification and targeting of G-quadruplex structures in MALAT1 long non-coding RNA. Nucleic Acids Res. 2022;50:397–410. 34904666 10.1093/nar/gkab1208 PMC8754639

[89] Ghosh A, et al. Identification of G-quadruplex structures in MALAT1 lncRNA that interact with nucleolin and nucleophosmin. Nucleic Acids Res. 2023;51:9415–31. 37558241 10.1093/nar/gkad639 PMC11314421

[90] Sahayasheela VJ, Sugiyama H. RNA G-quadruplex in functional regulation of noncoding RNA: challenges and emerging opportunities. Cell Chem Biol. 2024;31:53–70. 37909035 10.1016/j.chembiol.2023.08.010

[91] Wang W, et al. G-quadruplexes promote the motility in MAZ phase-separated condensates to activate CCND1 expression and contribute to hepatocarcinogenesis. Nat Commun. 2024;15:1045. 38316778 10.1038/s41467-024-45353-5 PMC10844655

[92] Mehta S, Zhang J. Liquid-liquid phase separation drives cellular function and dysfunction in cancer. Nat Rev Cancer. 2022;22:239–52. 35149762 10.1038/s41568-022-00444-7 PMC10036213

[93] Mimura M, et al. Quadruplex folding promotes the condensation of linker histones and DNAs via liquid–liquid phase separation. J Am Chem Soc. 2021;143:9849–57. 34152774 10.1021/jacs.1c03447

[94] Lee JJ, et al. Disruption of G-quadruplex dynamicity by BRCA2 abrogation instigates phase separation and break-induced replication at telomeres. Nucleic Acids Res. 2024;52:5756–73. 38587189 10.1093/nar/gkae251 PMC11162766

[95] Shil S, Tsuruta M, Kawauchi K, Miyoshi D. Factors affecting liquid-liquid phase separation of RGG peptides with DNA G-quadruplex. ChemMedChem. 2025;20:e202400460. 39256186 10.1002/cmdc.202400460

[96] Zhang Y, et al. G-quadruplex structures trigger RNA phase separation. Nucleic Acids Res. 2019;47:11746–54. 31722410 10.1093/nar/gkz978 PMC7145655

[97] Tsuruta M, et al. Controlling liquid–liquid phase separation of G-quadruplex-forming RNAs in a sequence-specific manner. Chem Commun (Camb). 2022;58:12931–4. 36321741 10.1039/d2cc04366a

[98] Robinson J, Raguseo F, Nuccio SP, Liano D, Di Antonio M. DNA G-quadruplex structures: more than simple roadblocks to transcription? Nucleic Acids Res. 2021;49:8419–31. 34255847 10.1093/nar/gkab609 PMC8421137

[99] Antariksa NF, Di Antonio M. The emerging roles of multimolecular G-quadruplexes in transcriptional regulation and chromatin organization. Acc Chem Res. 2024;57:3397–406. 39555660 10.1021/acs.accounts.4c00574 PMC11618987

[100] Paeschke K, Capra JA, Zakian VA. DNA replication through G-quadruplex motifs is promoted by the Saccharomyces cerevisiae Pif1 DNA helicase. Cell. 2011;145:678–91. 21620135 10.1016/j.cell.2011.04.015 PMC3129610

[101] Vannier JB, Pavicic-Kaltenbrunner V, Petalcorin MI, Ding H, Boulton SJ. RTEL1 dismantles T loops and counteracts telomeric G4-DNA to maintain telomere integrity. Cell. 2012;149:795–806. 22579284 10.1016/j.cell.2012.03.030

[102] Lin W, et al. Mammalian DNA2 helicase/nuclease cleaves G-quadruplex DNA and is required for telomere integrity. Embo J. 2013;32:1425–39. 23604072 10.1038/emboj.2013.88 PMC3655473

[103] Mohaghegh P, Karow JK, Brosh RM Jr., Bohr VA, Hickson ID. The Bloom’s and Werner’s syndrome proteins are DNA structure-specific helicases. Nucleic Acids Res. 2001;29:2843–9. 11433031 10.1093/nar/29.13.2843 PMC55766

[104] London TB, et al. FANCJ is a structure-specific DNA helicase associated with the maintenance of genomic G/C tracts. J Biol Chem. 2008;283:36132–9. 18978354 10.1074/jbc.M808152200 PMC2662291

[105] Lemmens B, van Schendel R, Tijsterman M. Mutagenic consequences of a single G-quadruplex demonstrate mitotic inheritance of DNA replication fork barriers. Nat Commun. 2015;6:8909. 26563448 10.1038/ncomms9909 PMC4654259

[106] Lerner LK, Sale JE. Replication of G quadruplex DNA. Genes (Basel). 2019. 10.3390/genes10020095. 30700033 10.3390/genes10020095 PMC6409989

[107] Sarkies P, Reams C, Simpson LJ, Sale JE. Epigenetic instability due to defective replication of structured DNA. Mol Cell. 2010;40:703–13. 21145480 10.1016/j.molcel.2010.11.009 PMC3145961

[108] Wang YH, et al. G4LDB 2.2: a database for discovering and studying G-quadruplex and i-Motif ligands. Nucleic Acids Res. 2022;50:D150-d160. 34718746 10.1093/nar/gkab952 PMC8728129

[109] Salvati E, et al. Telomere damage induced by the G-quadruplex ligand RHPS4 has an antitumor effect. J Clin Invest. 2007;117:3236–47. 17932567 10.1172/JCI32461 PMC2000812

[110] Burger AM, et al. The G-quadruplex-interactive molecule BRACO-19 inhibits tumor growth, consistent with telomere targeting and interference with telomerase function. Cancer Res. 2005;65:1489–96. 15735037 10.1158/0008-5472.CAN-04-2910

[111] Che T, et al. Discovery of novel Schizocommunin derivatives as telomeric G-quadruplex ligands that trigger telomere dysfunction and the deoxyribonucleic acid (DNA) damage response. J Med Chem. 2018;61:3436–53. 29618208 10.1021/acs.jmedchem.7b01615

[112] Rodriguez R, et al. A novel small molecule that alters shelterin integrity and triggers a DNA-damage response at telomeres. J Am Chem Soc. 2008;130:15758–9. 18975896 10.1021/ja805615w PMC2746963

[113] Konieczna N, et al. Telomerase inhibitor TMPyP4 alters adhesion and migration of breast-cancer cells MCF7 and MDA-MB-231. Int J Mol Sci. 2019. 10.3390/ijms20112670. 31151281 10.3390/ijms20112670 PMC6600420

[114] Grand CL, et al. The cationic porphyrin TMPyP4 down-regulates c-MYC and human telomerase reverse transcriptase expression and inhibits tumor growth in vivo. Mol Cancer Ther. 2002;1:565–73. 12479216

[115] Faudale M, Cogoi S, Xodo LE. Photoactivated cationic alkyl-substituted porphyrin binding to g4-RNA in the 5’-UTR of KRAS oncogene represses translation. Chem Commun (Camb). 2012;48:874–6. 22127206 10.1039/c1cc15850c

[116] Shen Z, et al. G-quadruplex stabilizer Tetra-Pt(bpy) disrupts telomere maintenance and impairs FAK-mediated migration of telomerase-positive cells. Int J Biol Macromol. 2022;213:858–70. 35697164 10.1016/j.ijbiomac.2022.06.015

[117] Tosoni B, et al. The G-quadruplex experimental drug QN-302 impairs liposarcoma cell growth by inhibiting MDM2 expression and restoring p53 levels. Nucleic Acids Res. 2025. 10.1093/nar/gkaf085. 39945321 10.1093/nar/gkaf085 PMC11822379

[118] Arshad T, Neidle S, Chandara S, Borazanci E. Early clinical experience with a novel first-in-class G-quadruplex experimental anti-cancer drug. Cancer Res. 2024;84:CT105.10.1158/1538-7445.AM2024-CT105.

[119] Wang XD, Lin JH, Hu MH. Discovery of a tribenzophenazine analog for binding to the KRAS mRNA G-quadruplex structures in the cisplatin-resistant non-small cell lung cancer. J Biol Chem. 2025;301:108164. 39793888 10.1016/j.jbc.2025.108164 PMC11847542

[120] Wang R, Zhang ZL, Hu MH. Diphenylaminothiophen-derived fluorescent ligands targeting mitochondrial DNA G-quadruplexes potentially for triple-negative breast cancer therapy. Int J Biol Macromol. 2025;302:140556. 39894118 10.1016/j.ijbiomac.2025.140556

[121] Wang XD, Liu YS, Liang ZL, Hu MH. Mitochondrial DNA-targeted triphenylamine-thiophene (TPATP)-derived ligands boost type-I/II photodynamic therapy for triple-negative breast cancer. Eur J Med Chem. 2025;289:117489. 40064143 10.1016/j.ejmech.2025.117489

[122] Wang XD, Liu YS, Chen MD, Hu MH. Discovery of a triphenylamine-based ligand that targets mitochondrial DNA G-quadruplexes and activates the cGAS-STING immunomodulatory pathway. Eur J Med Chem. 2024;269:116361. 38547736 10.1016/j.ejmech.2024.116361

[123] Huang F, et al. Small molecule as potent hepatocellular carcinoma progression inhibitor through stabilizing G-quadruplex DNA to activate replication stress responded DNA damage. Chem Biol Interact. 2025. 10.1016/j.cbi.2025.111469. 40057014 10.1016/j.cbi.2025.111469

[124] Xu H, et al. CX-5461 is a DNA G-quadruplex stabilizer with selective lethality in BRCA1/2 deficient tumours. Nat Commun. 2017;8:14432. 28211448 10.1038/ncomms14432 PMC5321743

[125] Jiang XC, et al. Discovery of a novel G-quadruplex and histone deacetylase (HDAC) dual-targeting agent for the treatment of triple-negative breast cancer. J Med Chem. 2022;65:12346–66. 36053318 10.1021/acs.jmedchem.2c01058

[126] Sanchez-Martin V, et al. The histone deacetylase inhibitor Scriptaid targets G-quadruplexes. Open Biol. 2025. 10.1098/rsob.240183. 39965659 10.1098/rsob.240183 PMC11835489

[127] Iachettini S, Biroccio A, Zizza P. Therapeutic use of G4-ligands in cancer: state-of-the-art and future perspectives. Pharmaceuticals (Basel). 2024. 10.3390/ph17060771. 38931438 10.3390/ph17060771 PMC11206494

[128] Teng FY, et al. G-quadruplex DNA: a novel target for drug design. Cell Mol Life Sci. 2021;78:6557–83. 34459951 10.1007/s00018-021-03921-8 PMC11072987

[129] Sun D, et al. Inhibition of human telomerase by a G-quadruplex-interactive compound. J Med Chem. 1997;40:2113–6. 9216827 10.1021/jm970199z

[130] Palma E, Carvalho J, Cruz C, Paulo A. Metal-based G-quadruplex binders for cancer theranostics. Pharmaceuticals (Basel). 2021. 10.3390/ph14070605. 34201682 10.3390/ph14070605 PMC8308583

[131] Zheng XH, et al. A cisplatin derivative tetra-Pt(bpy) as an oncotherapeutic agent for targeting ALT cancer. J Natl Cancer Inst. 2017;109. 10.1093/jnci/djx061 28521363

[132] Hu MH, Wu TY, Huang Q, Jin G. New substituted quinoxalines inhibit triple-negative breast cancer by specifically downregulating the c-MYC transcription. Nucleic Acids Res. 2019;47:10529–42. 31584090 10.1093/nar/gkz835 PMC6846596

[133] Hu MH, et al. Discovery of a new four-leaf clover-like ligand as a potent c-MYC transcription inhibitor specifically targeting the promoter G-quadruplex. J Med Chem. 2018;61:2447–59. 29474069 10.1021/acs.jmedchem.7b01697

[134] Dutta D, et al. Cell penetrating thiazole peptides inhibit c-MYC expression via site-specific targeting of c-MYC G-quadruplex. Nucleic Acids Res. 2018;46:5355–65. 29762718 10.1093/nar/gky385 PMC6009605

[135] Nambiar M, et al. Formation of a G-quadruplex at the BCL2 major breakpoint region of the t(14;18) translocation in follicular lymphoma. Nucleic Acids Res. 2011;39:936–48. 20880994 10.1093/nar/gkq824 PMC3035451

[136] Pandya N, Singh M, Rani R, Kumar V, Kumar A. G-quadruplex-mediated specific recognition, stabilization and transcriptional repression of bcl-2 by small molecule. Arch Biochem Biophys. 2023;734:109483. 36513132 10.1016/j.abb.2022.109483

[137] Salvati E, et al. Evidence for G-quadruplex in the promoter of vegfr-2 and its targeting to inhibit tumor angiogenesis. Nucleic Acids Res. 2014;42:2945–57. 24335081 10.1093/nar/gkt1289 PMC3950687

[138] Long W, et al. Targeting hTERT promoter G-quadruplex DNA structures with small-molecule ligand to downregulate hTERT expression for triple-negative breast cancer therapy. J Med Chem. 2024;67:13363–82. 38987863 10.1021/acs.jmedchem.4c01255

[139] Collie GW, Parkinson GN. The application of DNA and RNA G-quadruplexes to therapeutic medicines. Chem Soc Rev. 2011;40:5867–92. 21789296 10.1039/c1cs15067g

[140] Katsuda Y, et al. A small molecule that represses translation of G-quadruplex-containing mRNA. J Am Chem Soc. 2016;138:9037–40. 27410677 10.1021/jacs.6b04506

[141] Miglietta G, et al. RNA G-quadruplexes in kirsten ras (KRAS) oncogene as targets for small molecules inhibiting translation. J Med Chem. 2017;60:9448–61. 29140695 10.1021/acs.jmedchem.7b00622

[142] Li ML, et al. Discovery of novel coumarin-quinolinium derivatives as Pan-KRAS translation inhibitors by targeting 5’-UTR RNA G-quadruplexes. J Med Chem. 2024;67:1961–81. 38272464 10.1021/acs.jmedchem.3c01773

[143] Kawauchi K, et al. An anionic phthalocyanine decreases NRAS expression by breaking down its RNA G-quadruplex. Nat Commun. 2018;9:2271. 29891945 10.1038/s41467-018-04771-y PMC5995912

[144] Le Sénéchal R, et al. Alternative splicing of BCL-x is controlled by RBM25 binding to a G-quadruplex in BCL-x pre-mRNA. Nucleic Acids Res. 2023;51:11239–57. 37811881 10.1093/nar/gkad772 PMC10639069

[145] Bryan TM. Mechanisms of DNA replication and repair: insights from the study of G-quadruplexes. Molecules. 2019. 10.3390/molecules24193439. 31546714 10.3390/molecules24193439 PMC6804030

[146] De Magis A, et al. DNA damage and genome instability by G-quadruplex ligands are mediated by R loops in human cancer cells. Proc Natl Acad Sci USA. 2019;116:816–25. 30591567 10.1073/pnas.1810409116 PMC6338839

[147] Huang F, et al. Small molecule as potent hepatocellular carcinoma progression inhibitor through stabilizing G-quadruplex DNA to activate replication stress responded DNA damage. Chem Biol Interact. 2025;412:111469. 40057014 10.1016/j.cbi.2025.111469

[148] McLuckie KI, et al. G-quadruplex DNA as a molecular target for induced synthetic lethality in cancer cells. J Am Chem Soc. 2013;135:9640–3. 23782415 10.1021/ja404868t PMC3964824

[149] Kaelin WG Jr. The concept of synthetic lethality in the context of anticancer therapy. Nat Rev Cancer. 2005;5:689–98. 16110319 10.1038/nrc1691

[150] Bryant HE, et al. Specific killing of BRCA2-deficient tumours with inhibitors of poly(ADP-ribose) polymerase. Nature. 2005;434:913–7. 15829966 10.1038/nature03443

[151] Farmer H, et al. Targeting the DNA repair defect in BRCA mutant cells as a therapeutic strategy. Nature. 2005;434:917–21. 15829967 10.1038/nature03445

[152] Li C, Xue Y, Ba X, Wang R. The role of 8-oxoG repair systems in tumorigenesis and cancer therapy. Cells. 2022. 10.3390/cells11233798. 36497058 10.3390/cells11233798 PMC9735852

[153] Soldatenkov VA, Vetcher AA, Duka T, Ladame S. First evidence of a functional interaction between DNA quadruplexes and poly(ADP-ribose) polymerase-1. ACS Chem Biol. 2008;3:214–9. 18338862 10.1021/cb700234f

[154] Salvati E, et al. PARP1 is activated at telomeres upon G4 stabilization: possible target for telomere-based therapy. Oncogene. 2010;29:6280–93. 20802516 10.1038/onc.2010.344

[155] Edwards AD, Marecki JC, Byrd AK, Gao J, Raney KD. G-quadruplex loops regulate PARP-1 enzymatic activation. Nucleic Acids Res. 2021;49:416–31. 33313902 10.1093/nar/gkaa1172 PMC7797039

[156] Sengar A, et al. Structure of a (3+1) hybrid G-quadruplex in the PARP1 promoter. Nucleic Acids Res. 2019;47:1564–72. 30551210 10.1093/nar/gky1179 PMC6379715

[157] Hilton J, et al. Results of the phase I CCTG IND.231 trial of CX-5461 in patients with advanced solid tumors enriched for DNA-repair deficiencies. Nat Commun. 2022;13:3607. 35750695 10.1038/s41467-022-31199-2 PMC9232501

[158] Zimmer J, et al. Targeting BRCA1 and BRCA2 deficiencies with G-quadruplex-interacting compounds. Mol Cell. 2016;61:449–60. 26748828 10.1016/j.molcel.2015.12.004 PMC4747901

[159] Salvati E, et al. Lead discovery of dual G-quadruplex stabilizers and poly(ADP-ribose) polymerases (PARPs) inhibitors: a new avenue in anticancer treatment. J Med Chem. 2017;60:3626–35. 28445046 10.1021/acs.jmedchem.6b01563

[160] Day TA, et al. PARP3 is a promoter of chromosomal rearrangements and limits G4 DNA. Nat Commun. 2017;8:15110. 28447610 10.1038/ncomms15110 PMC5414184

[161] Yan J, et al. Pyridine-bis(benzimidazole) induces DNA damage at G-quadruplex loci and promotes synthetic lethality with DNA repair inhibition. Nucleic Acids Res. 2025. 10.1093/nar/gkaf543. 40568940 10.1093/nar/gkaf543 PMC12199159

[162] Masud T, et al. Ubiquitin-mediated DNA damage response is synthetic lethal with G-quadruplex stabilizer CX-5461. Sci Rep. 2021;11:9812. 33963218 10.1038/s41598-021-88988-w PMC8105411

[163] Dharmaiah S, et al. G-quadruplex stabilizer CX-5461 effectively combines with radiotherapy to target α-thalassemia/mental retardation X-linked-deficient malignant glioma. Neuro Oncol. 2025;27:932–47. 39570009 10.1093/neuonc/noae248 PMC12083236

[164] Ahmed AA, Neidle S. A G-quadruplex-binding small molecule and the HDAC inhibitor SAHA (Vorinostat) act synergistically in gemcitabine-sensitive and resistant pancreatic cancer cells. Molecules. 2020. 10.3390/molecules25225407. 33227941 10.3390/molecules25225407 PMC7699281

[165] Sanchez-Martin V, et al. The histone deacetylase inhibitor Scriptaid targets G-quadruplexes. Open Biol. 2025;15:240183. 39965659 10.1098/rsob.240183 PMC11835489

[166] Li P, et al. Targeting G-quadruplex by TMPyP4 for inhibition of colorectal cancer through cell cycle arrest and boosting anti-tumor immunity. Cell Death Dis. 2024;15:816. 39528472 10.1038/s41419-024-07215-2 PMC11554887

[167] Miglietta G, Russo M, Duardo RC, Capranico G. G-quadruplex binders as cytostatic modulators of innate immune genes in cancer cells. Nucleic Acids Res. 2021;49:6673–86. 34139015 10.1093/nar/gkab500 PMC8266585

[168] Miglietta G, Marinello J, Russo M, Capranico G. Ligands stimulating antitumour immunity as the next G-quadruplex challenge. Mol Cancer. 2022;21:180. 36114513 10.1186/s12943-022-01649-y PMC9482198

[169] Marzano S, et al. Balancing affinity, selectivity, and cytotoxicity of hydrazone-based G-quadruplex ligands for activation of interferon β genes in cancer cells. J Med Chem. 2022;65:12055–67. 36074772 10.1021/acs.jmedchem.2c00772 PMC9511478

[170] Leonetti C, et al. G-quadruplex ligand RHPS4 potentiates the antitumor activity of camptothecins in preclinical models of solid tumors. Clin Cancer Res. 2008;14:7284–91. 19010844 10.1158/1078-0432.CCR-08-0941

[171] Biffi G, Tannahill D, McCafferty J, Balasubramanian S. Quantitative visualization of DNA G-quadruplex structures in human cells. Nat Chem. 2013;5:182–6. 23422559 10.1038/nchem.1548 PMC3622242

[172] Doimo M, et al. Enhanced mitochondrial G-quadruplex formation impedes replication fork progression leading to mtDNA loss in human cells. Nucleic Acids Res. 2023;51:7392–408. 37351621 10.1093/nar/gkad535 PMC10415151

[173] Hu MH. Molecular engineering of a near-infrared fluorescent ligand for tracking mitochondrial DNA G-quadruplexes. Anal Chim Acta. 2021;1169:338600. 34088367 10.1016/j.aca.2021.338600

[174] Chen XC, et al. Monitoring and modulating mtDNA G-quadruplex dynamics reveal its close relationship to cell glycolysis. J Am Chem Soc. 2021;143:20779–91. 34865478 10.1021/jacs.1c08860

[175] Guo X, et al. An organic molecular compound for in situ identification of mitochondrial G-quadruplexes in live cells. J Mater Chem B. 2022;10:430–7. 34940779 10.1039/d1tb02296b

[176] Huang WC, et al. Direct evidence of mitochondrial G-quadruplex DNA by using fluorescent anti-cancer agents. Nucleic Acids Res. 2015;43:10102–13. 26487635 10.1093/nar/gkv1061 PMC4666356

[177] Schultz CW, et al. The FDA-approved anthelmintic pyrvinium pamoate inhibits pancreatic cancer cells in nutrient-depleted conditions by targeting the mitochondria. Mol Cancer Ther. 2021;20:2166–76. 34413127 10.1158/1535-7163.MCT-20-0652 PMC8859979

[178] Ahmed AA, et al. The potent G-quadruplex-binding compound QN-302 downregulates S100P gene expression in cells and in an in vivo model of pancreatic cancer. Molecules. 2023. 10.3390/molecules28062452. 36985425 10.3390/molecules28062452 PMC10051992

[179] Figueiredo J, Mergny JL, Cruz C. G-quadruplex ligands in cancer therapy: progress, challenges, and clinical perspectives. Life Sci. 2024;340:122481. 38301873 10.1016/j.lfs.2024.122481

[180] Sullivan HJ, Chen B, Wu C. Molecular dynamics study on the binding of an anticancer DNA G-quadruplex stabilizer, CX-5461, to human telomeric, c-KIT1, and c-Myc G-quadruplexes and a DNA duplex. J Chem Inf Model. 2020;60:5203–24. 32820923 10.1021/acs.jcim.0c00632

[181] Savva L, Georgiades SN. Recent developments in small-molecule ligands of medicinal relevance for harnessing the anticancer potential of G-quadruplexes. Molecules. 2021. 10.3390/molecules26040841. 33562720 10.3390/molecules26040841 PMC7914483

[182] Abrahamsson A, et al. Linker design principles for the precision targeting of oncogenic G-quadruplex DNA with G4-ligand-conjugated oligonucleotides. Bioconjug Chem. 2025;36:724–36. 40112195 10.1021/acs.bioconjchem.5c00008 PMC12006964

[183] Chen L, Dickerhoff J, Sakai S, Yang D. DNA G-quadruplex in human telomeres and oncogene promoters: structures, functions, and small molecule targeting. Acc Chem Res. 2022;55:2628–46. 36054116 10.1021/acs.accounts.2c00337 PMC9937053

[184] Bidzinska J, Cimino-Reale G, Zaffaroni N, Folini M. G-quadruplex structures in the human genome as novel therapeutic targets. Molecules. 2013;18:12368–95. 24108400 10.3390/molecules181012368 PMC6270421

[185] Biver T. Discriminating between parallel, anti-parallel and hybrid G-quadruplexes: mechanistic details on their binding to small molecules. Molecules. 2022. 10.3390/molecules27134165. 35807410 10.3390/molecules27134165 PMC9268745

[186] Berner A, et al. G4-ligand-conjugated oligonucleotides mediate selective binding and stabilization of individual G4 DNA structures. J Am Chem Soc. 2024;146:6926–35. 38430200 10.1021/jacs.3c14408 PMC10941181

[187] Cadoni E, et al. A red light-triggered chemical tool for sequence-specific alkylation of G-quadruplex and I-motif DNA. Nucleic Acids Res. 2023;51:4112–25. 36971129 10.1093/nar/gkad189 PMC10201448

[188] Tassinari M, et al. Selective targeting of mutually exclusive DNA G-quadruplexes: HIV-1 LTR as paradigmatic model. Nucleic Acids Res. 2020;48:4627–42. 32282912 10.1093/nar/gkaa186 PMC7229848

[189] Nuccio SP, et al. Chemically modified CRISPR-Cas9 enables targeting of individual G-quadruplex and i-motif structures, revealing ligand-dependent transcriptional perturbation. bioRxiv. 2025. 10.1101/2024.10.14.618195. 41366211 10.1038/s41467-025-67074-z PMC12796454

[190] Qin G, et al. Targeting specific DNA G-quadruplexes with CRISPR-guided G-quadruplex-binding proteins and ligands. Nat Cell Biol. 2024;26:1212–24. 38961283 10.1038/s41556-024-01448-1

